# Quantifying the relative immune cell activation from whole tissue/organ-derived differentially expressed gene data

**DOI:** 10.1038/s41598-017-12970-8

**Published:** 2017-10-09

**Authors:** Edward Wijaya, Yoshinobu Igarashi, Noriyuki Nakatsu, Yasunari Haseda, Joel Billaud, Yi-An Chen, Kenji Mizuguchi, Hiroshi Yamada, Ken Ishii, Taiki Aoshi

**Affiliations:** 1 System Immunology Laboratory, Immunology Frontier Research Centre, Osaka University, Osaka, 565-0781 Japan; 2 Department of Genome Informatics, Research Institute for Microbial Diseases, Osaka University, Osaka, 565-0781 Japan; 3 Toxicogenomics-Informatics Project, National Institutes of Biomedical Innovation, Health and Nutrition, Osaka, 567-0085 Japan; 4 Bioinformatics Project, National Institutes of Biomedical Innovation, Health and Nutrition, Osaka, 567-0085 Japan; 5 Vaccine Science Laboratory, Immunology Frontier Research Centre, Osaka University, Osaka, 565-0781 Japan; 6 Laboratory of Adjuvant Innovation, National Institutes of Biomedical Innovation, Health and Nutrition, Osaka, 567-0085 Japan; 7 Vaccine Dynamics Project, BIKEN Innovative Vaccine Research Alliance Laboratories, Osaka University, Osaka, 565-0871 Japan

## Abstract

Evaluation of immune responses in individual immune cell types is important for the development of new medicines. Here, we propose a computational method designated ICEPOP (Immune CEll POPulation) to estimate individual immune cell type responses from bulk tissue and organ samples. The relative gene responses are scored for each cell type by using the data from differentially expressed genes derived from control- vs drug-treated sample pairs, and the data from public databases including ImmGen and IRIS, which contain gene expression profiles of a variety of immune cells. By ICEPOP, we analysed cell responses induced by vaccine-adjuvants in the mouse spleen, and extended the analyses to human peripheral blood mononuclear cells and gut biopsy samples focusing on human papilloma virus vaccination and inflammatory bowel disease treatment with Infliximab. In both mouse and human datasets, our method reliably quantified the responding immune cell types and provided insightful information, demonstrating that our method is useful to evaluate immune responses from bulk sample-derived gene expression data. ICEPOP is available as an interactive web site (https://vdynamics.shinyapps.io/icepop/) and Python package (https://github.com/ewijaya/icepop).

## Introduction

The immune system consists of many different types of cells, which function in concert to mediate immune responses. However, the interaction complexity hinders the evaluation of the individual immune cell type responses to drug treatment. For example, especially in a bulk sample like a whole organ or biopsy sample, the evaluation requires *in vitro* analysis of each isolated immune cell type, which is usually effortful.

Recent large-scale multi-omics technologies have accelerated the development of new *in silico* methods to understand complex immune responses^1,2^ and immune-drug interactions^3^. The CTen (Cell Type Enrichment)^4^ and GSEA (Gene Set Enrichment Analysis)^5^ programs have been used to analyse immune cell responses using large-scale gene expression data. These methods calculate enrichment scores according to the presence of the query genes over the gene set references. Various defined gene sets are available and have been widely used for a variety of biological analyses including immune responses.

These methods use the annotation enrichment of various biological processes for their primary output. They usually do not account for gene expression information in their calculations. The methods are qualitative and hence are not suitable for quantitative estimation analyses, especially of immune cell responses from whole-organ or tissue-derived samples, because whole samples usually contain many different cell types. In cases where a single cell population responds specifically to drug treatment, these qualitative methods are also practical for sample characterisation because most of the gene responses derive from a single cell population (see Supplementary Fig. S14a). In other cases, different cell populations respond simultaneously to drug treatment. In such cases, CTen and GSEA only provide a mixture of annotations from the different cell populations together; these mixed qualitative results are usually difficult to interpret, especially to identify the key responding cell population(s) (see Supplementary Fig. S14b–d and Supplementary Fig. S15).

Whole tissue/organ sample microarray analysis also poses a technical hindrance. During the sample preparation, the whole organ samples can be contaminated with neighbouring organs or tissues. In the ongoing *Adjuvant Database Project* (http://adjuvantdb.nibiohn.go.jp), we have sporadically experienced those unexpected contaminations despite very careful handling. In such cases, the contaminants are unavoidably co-purified with the primary target RNA and introduce detrimental false results in the gene expression analyses.

**Figure 1:**
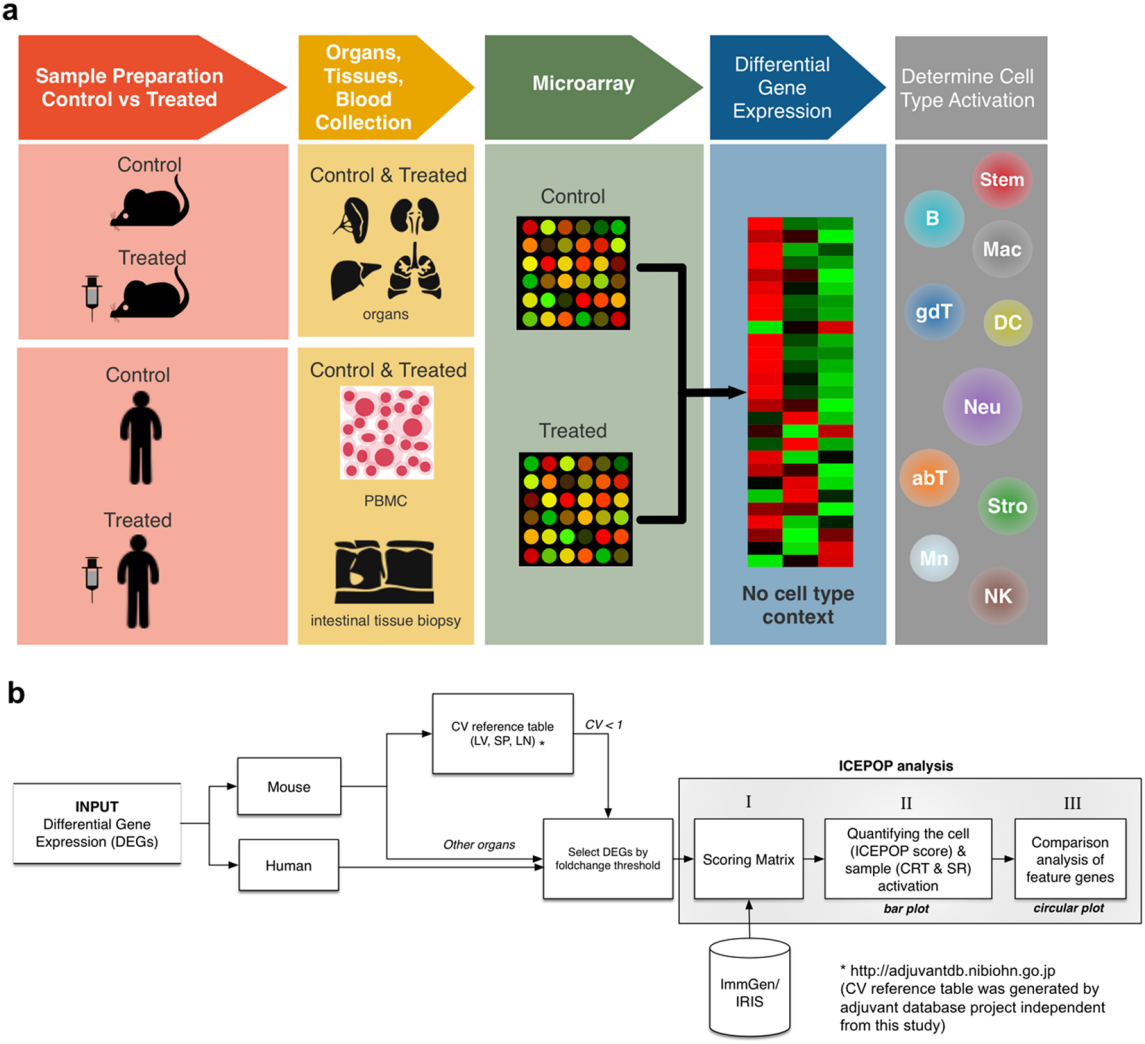
Sample and data processing scheme in ICEPOP analysis. (**a**) Sample processing scheme of quantifying immune cell activation from tissue- or organ-derived differential expression genes (DEGs). RNA is purified from a whole organ (e.g. spleen) or PBMC, and then gene expression is measured by microarray. Typical analysis requires control and treated sample pairs, and then their DEGs are used to determine the immune cell activation. (**b**) Data processing framework of ICEPOP analysis. First, species need to be specified. For sample derived from mouse liver, spleen, or lymph node, CV filter is available. For ICEPOP analysis, the user needs to set the fold-change threshold to select the actual input DEGs. ICEPOP calculates the relative activation score for each cell type using a scoring matrix, and then produces a bar graph and circular plot as outputs. Roman numerals (I, II, III) refer to the processes described in Supplementary Figure S2.

To solve these problems, we have developed a computational program designated ICEPOP (Immune CEll POPulation) to quantitatively estimate the relative immune cell response for each cell type. ICEPOP uses gene names and the associated expression values from microarray data of mouse and human organ or tissue samples (Fig. 1a). Based on the given gene expression fold-changes and names, ICEPOP calculates the relative response scores for each immune cell type defined by the reference matrix made from public gene expression databases including the ImmGen^6^ (for mouse) and the IRIS^7^ (for human), which encompass a wide-range of immunological cell types and states (Fig. 1b). ICEPOP is intended to analyse non-purified bulky samples such as whole organs or biopsy tissue. We integrated a nearby organ-derived gene contamination removing filter. ICEPOP analysis provides two major outputs: (i) bar graph presentation of the relative response score for ImmGen or IRIS defined immune cell types; and (ii) comparison of differently treated samples with circular map presentation, allowing more detailed analysis and interpretation.

In this study, we tested ICEPOP on adjuvant administration studies in mice and two human studies including vaccination of virus-like particles (VLPs) based on human papilloma virus (HPV), and infliximab treatment of inflammatory bowel disease. Insightful characterizations of the immune cell responses are described.

## Results and Discussion

### Characterization of immune cell activation by adjuvants in mouse spleen

ICEPOP was first tested on two publicly available microarray datasets—GSE63332 and GSE7768—from the Gene Expression Omnibus (GEO) repository (https://www.ncbi.nlm.nih.gov/geo/) (Table 1). These data were derived from mouse spleen, normalized as previously described^8,9^. Contaminating genes from nearby organs, such as the pancreas, were removed by a coefficient of variation (*CV*) *filter* (see Methods and Fig. 1b). Then, activation of each cell type was quantified by ICEPOP with several analytical parameters (see Methods).

**Table 1:** Datasets used in this study.

Study	Treatment	Samples	Dosage	Organs	Organism	Reference
GSE63332	With bCD, 6 hours after i.d. and i.p. injection	3 (PBS, i.d.) 3 (bCD, i.d.) 3 (PBS, i.p.) 3 (bCD, i.p.)	300 μg (bCD)	Spleen	*Mus musculus*	8
GSE7768	With LPS and MPL, 6 hours after i.v. injection	3 (PBS, i.v.) 3 (LPS, i.v.) 3 (MPL, i.v.)	10 μg (LPS) 30 μg (MPL)	Spleen	*Mus musculus*	9
GSE13587	With placebo and HPV-16-L1 VLP on cultured cells	7 (placebo) and 20 (vaccine)	2.5 μg (VLP)	PBMC	*Homo Sapiens*	13
GSE16879	With IFX infusion in patient with inflammatory bowel disease: UC and CD	12 (healthy) and 61 (disease)	2.0 μg (IFX)	Colon and Ileum mucosa	*Homo Sapiens*	21

Abbreviations: bCD: hydroxypropyl-β-cyclodextrin; PBS: phosphate buffered saline; i.d.; intradermal; i.p.: intraperitoneal; i.v.: intravenous; LPS: lipopolysaccharide; MPL: monophosphoryl lipid A; HPV; human papillomavirus; VLP: virus-like particle; IFX: infliximab; UC: ulcerative colitis; CD: Crohn’s disease.

#### GSE63332 dataset

Examination of the GSE63332 dataset (Table 1) focused on hydroxypropyl-β-cyclodextrin (bCD). bCD is a potent vaccine adjuvant in mice^8^. Local high concentrations of bCD temporally damage host cells at the administration site. The damaged cells release DNA, which is key in bCD’s adjuvant character^8^. In this dataset, mice received phosphate buffered saline (PBS) or bCD via intradermal (ID) or intraperitoneal (IP) injection. Six hours later the spleen was removed for microarray analysis of gene expressions. Differentially expressed genes (DEGs) for paired PBS and bCD injected samples were compared (Supplementary Fig. S1a).

**Figure 2:**
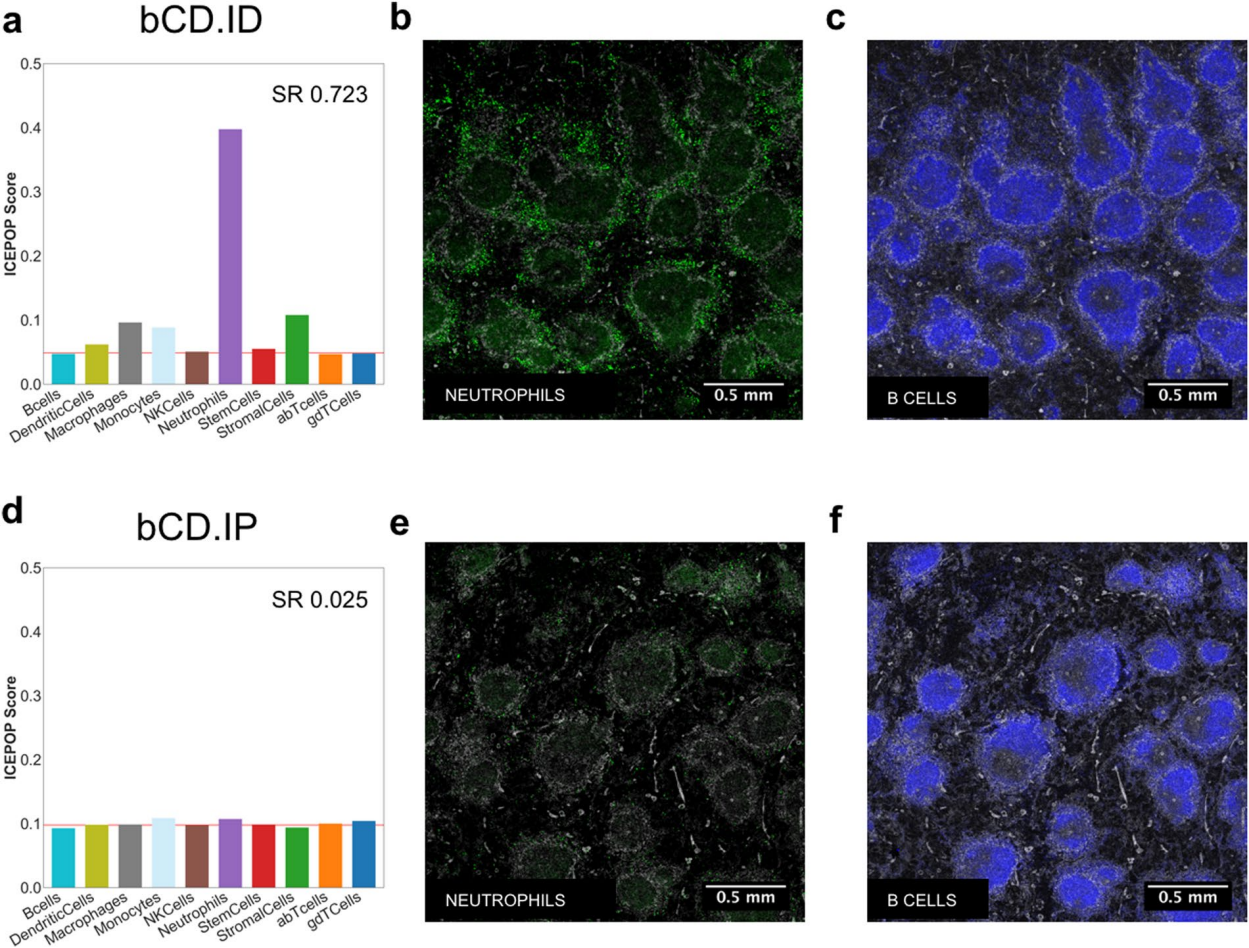
Immune cell population responses in the GSE63332 dataset. ICEPOP score (y-axis), sample response (SR) (inlet), and cell type response threshold (CRT) (horizontal red line) are shown in the bar graph of (**a**) ID and (**d**) IP bCD-treated spleens. Immunofluorescent staining of the spleen section via ID (**b,c**) or IP (**d,f**) administration of bCD for Neutrophil (**b,e**), and B cells (**c,f**) markers. Activated neutrophils is indicated by a stronger green staining in (**b**) compared with (**f**).

In bCD.ID samples, neutrophils showed the highest ICEPOP score among 10 different cell types (Fig. 2a), implicating neutrophils as the most activated cell type in the spleen in this condition. A bar plot of ICEPOP analysis quantitatively presents three analytical scores (see Methods for detailed descriptions). The ICEPOP score represents the relative gene response in each cell type (y-axis of the bar graph). The cell type response threshold (CRT) indicated by the red horizontal line represents the threshold value of cell type response. For example, in bCD.ID, the CRT was 0.049. The ICEPOP scores of six cell types including dendritic cells (ICEPOP score = 0.062), macrophages (0.096), monocytes (0.088), natural killer (NK) cells (0.050), neutrophils (0.398), stem cells (0.055), and stromal cells (0.108) were higher than this CRT, the latter two barely, indicating that the cells responded to bCD ID injection. With the same criteria, B cells, alpha beta T cells, and gamma delta T cells did not respond (Fig. 2a). The third analytical score, the sample response (SR) score, represents the overall sample response (in this case, whole spleen). Based on our null data simulation (Supplementary Fig. S13b, discussed later), an SR score exceeding about 0.3 in the mouse spleen indicates that the sample is substantially responding to the drug. In bCD.ID, SR was 0.723, suggesting bCD ID injection induced considerable biological responses in the spleen (Fig. 2a).

In contrast, in the bCD.IP condition, ICEPOP scores of all cell types and CRT were about 0.1 and 0.098, respectively, indicating virtually no responses (Fig. 2d). The SR of 0.025 also indicated no response to the IP route of bCD administration. Of note, the CRT value almost equaled the theoretical maximum (0.100) of the 10 cell type analysis (see Methods for more detailed description), bolstering the lack of detectable immunological cell responses.

The difference between administration routes was confirmed by histology examination. In the spleen cryosections stained with markers of neutrophils (Ly-6G) and B cells (B220), Ly-6G staining was prominent in bCD.ID (Fig. 2b) but not in bCD.IP (Fig. 2e), indicating neutrophils were activated in the spleen 6 hours after bCD ID administration, but not after IP administration. Correspondingly, there was no difference in B cell staining after these two treatments (Fig. 2c and f). The results were consistent with ICEPOP analysis (Fig. 2a and d). The collective results demonstrated that ICEPOP analysis can provide reliable evaluation of the sample responses at both cell type and whole organ levels.

#### GSE7768 dataset

**Figure 3:**
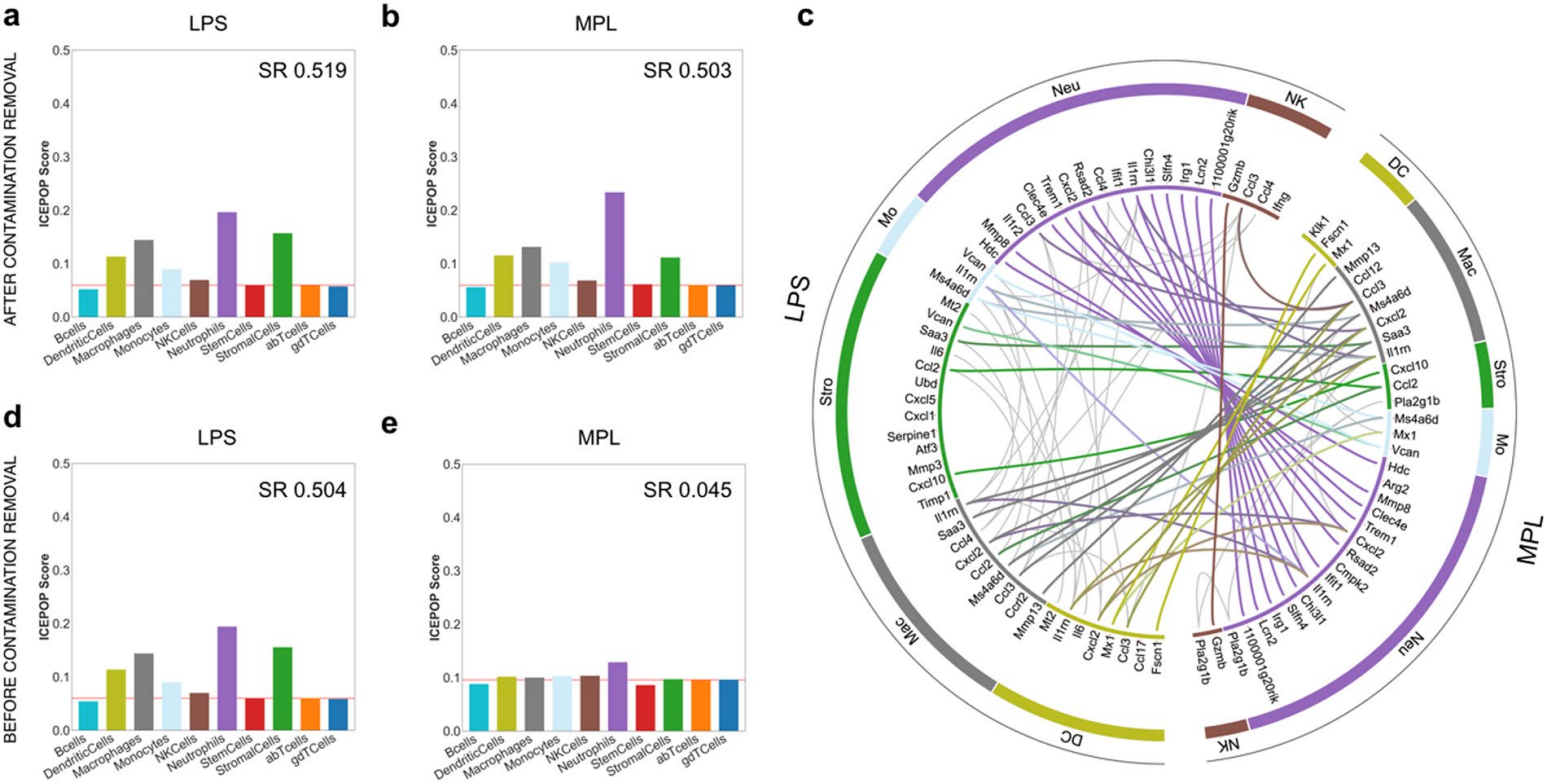
Immune cell population responses in the GSE7768 dataset. Bar graph presentation of ICEPOP score for each cell type after LPS (**a,d**) or MPL (**b,e**) administration. Immune cell responses (**a,b**) with or without (**d,e**) CV filtering of contaminated gene removal are shown. The horizontal red line indicates the CRT. Scores in the panels are sample response (SR) scores. (**c**) The circular plot of immune cell responses of (**a**) vs (**b**). The genes are linked with coloured thick lines if they coexist in different samples.

The examination of the GSE7768 compared lipopolysaccharide (LPS) and monophosphoryl Lipid A (MPL) 6 hours after injection (Table 1). LPS is a prototypic component of the Gram-negative bacterial cell wall. It can be toxic. MPL is a derivative of LPS with low toxicity^9,10^. In this dataset^9^, administrations were all performed via intravenous (IV) injection. The PBS, LPS, and MPL groups each comprised three mice; data from the total of nine spleens were used for ICEPOP analysis. DEGs were calculated as PBS vs LPS or PBS vs MPL (Supplementary Fig. S1b). The CV filter (described in later section) was applied and cell type responses evaluated. With the LPS treatment (Fig. 3a), six of ten cell types (dendritic cells, macrophages, monocytes, NK cells, neutrophils, and stromal cells) surpassed the CRT (0.060), indicating LPS responsiveness. The same cell types responded to MPL with CRT of 0.060 (Fig. 3b). Stromal cells showed a stronger response to LPS than to MPL (Fig. 3a vs b).

### Feature gene analysis in each activated cell type

We further evaluated the gene differences between LPS and MPL samples using the gene level response score of ICEPOP (Supplementary Fig. S2e,m; see Methods for detail). This score was obtained for all genes, and the top 100 scoring genes were identified (Supplementary Fig. S2n). These genes and cell type paired information were plotted in a circular plot (Fig. 3c). In the plot, some genes appeared more than once, since many genes are expressed by multiple cell populations; in the plot, these co-occurring genes are linked with lines. Coloured lines indicate that they appeared in both LPS and MPL samples, whereas those appearing in LPS or MPL only are denoted by grey lines. This circular plot strongly indicated that many of the feature genes with a relatively high gene level response score were in both sample types, suggesting that LPS and MPL induce very similar gene responses in the mouse spleen. On the other hand, LPS induced stronger gene responses in stromal cells for *Ubd, Cxcl5, Cxcl1, Serpine1, Atf3, Mmp3*, and *Timp1*, while dendritic cells, macrophages, monocytes, neutrophils, and NK cells showed similar gene responses to LPS and MPL (Fig. 3c). We are unaware of any prior report of the different sensitivity of stromal cells toward LPS and MPL; this may explain the lower biological toxicity of MPL.

### Effect of gene contamination from other organs on ICEPOP analysis

Microarray analysis is highly susceptible to contamination. When harvesting the spleen, fragments of neighbouring tissues including the pancreas and duodenum might be included. We observed heavy contamination of at least two MPL data of GSE7768 with pancreas- and duodenum-derived genes, which detrimentally affected the analysis (Fig. 3b vs e).

To lessen the effect of contaminated tissue-derived genes *in silico*, we applied our originally developed coefficient of variation (CV) reference table (see Fig. 1b). CV is widely used to compare the amount of variation of one variable^11^. Higher CV in the microarray data of multiple replicates indicates greater dispersion of gene expression among replicates. Therefore, genes with high CV values in control (PBS-treated) samples were more likely to originate from other, sporadically contaminating tissues. To calculate the CV for each gene, we utilized samples of the ongoing Adjuvant Database Project, which contains microarray expression data from PBS-treated control mouse spleen (33 samples), liver (33 samples), and lymph nodes (18 samples) (unpublished data). By analysing these samples, we empirically determined that the genes with a CV value exceeding 1 were more likely to be derived from tissue contamination. This control sample derived CV values strongly correlated with entropy scores of pancreas or intestine in BARCODE 3.0^12^, suggesting that high CV genes are likely from pancreatic or intestinal (duodenum and small intestine) tissues (Supplementary Fig. S3). The same BARCODE analysis of liver and lymph node control samples was not as evocative as spleen (Supplementary Fig. S3), but removing those high-CV genes from the following analysis would expect to stabilize the analysis result, especially for expression value utilizing analysis like ICEPOP. Subsequently, we integrated this ‘CV more than 1 cut-filter’ in ICEPOP as default when the CV reference table is available (see Fig. 1b).

For the GSE7768 contamination analysis, we plotted the gene expression data (x-axis) together with our reference CV values (y-axis) for all nine samples (Supplementary Fig. S4). MPL samples in the third row, especially the first and second replicates, displayed dots at the upper right areas of the plot, and the other samples did not (Supplementary Fig. S4). These genes with high expression and high CV were considered a signal from the contaminating tissues in this dataset. To validate the reliability, we generated a gene expression heat map of these presumed signal genes. We also placed a heat map of tissue specificity entropy score obtained from the BARCODE 3.0 database^12^, and placed them side-by-side (Supplementary Fig. S5). Most of the genes with high expression and high CV were strongly correlated with the genes derived from pancreatic tissues (pancreas and Islets of Langerhans) and intestinal tissues (duodenum, small intestine, jejunum, ileum, colon, intestinal epithelial cells and caecal tissue). Although not all of the high-CV genes were derived from contaminated tissues (Klk1 and Ighg1 were expressed in all 9 samples), most of the high-CV genes were correlated with pancreas- and duodenum-derived genes. In addition, the same plot of bCD samples (Supplementary Fig. S6a) indicated that they were free of detectable contamination, and their high-CV genes (upper left area in the graph) correlated mostly with spleen, as with the bCD samples (Supplementary Fig. S6b). These results strongly suggest that our CV filtering method is a reliable approach to remove contaminated genes *in silico*.

The contaminated genes unusually caused high fold-changes and profoundly affected the ICEPOP analysis, especially GSE7768 analysis. With or without of the contamination removal CV filter, the SR of the LPS did not change (before CV filter = 0.504 and after = 0.519) indicating that LPS was not contaminated (Fig. 3a vs d). However, in the MPL, without contamination removal, ICEPOP analysis resulted in almost no response with SR of 0.045 (Fig. 3e). Interestingly, with CV filtering, the same MPL-treated spleen data showed clear responses with SR of 0.503 (Fig. 3b), very similar to that of LPS (Fig. 3a). Taken together, these results confirmed the effectiveness of our CV filtering method, and showed that removing the contaminated tissue-derived genes provides more reliable results.

### ICEPOP analysis of human PBMC data from HPV-16L1 VLP vaccine

We also applied our method to human samples, in this case a peripheral blood mononuclear cell (PBMC) microarray dataset from the HPV-16L1 VLP (HPV-VLP) vaccine study^13^. This dataset (GSE13587) was also obtained from the GEO repository (Table 1). It contains gene expression microarray data of 4 groups: either placebo or HPV-VLP vaccine injection, at two-different times: pre*-* and post*-* (see Supplementary Fig. S1c). For the microarray analysis, PBMCs were stimulated *in vitro* with either medium or HPV-VLP in this dataset. We calculated the DEGs for medium vs HPV-VLP pairs, producing 4 input DEG data including pre-placebo, post-placebo, pre-vaccine, post-vaccine. This dataset focused on the induced genes difference after *in vitro* stimulation with HPV-VLP in these groups.

**Figure 4:**
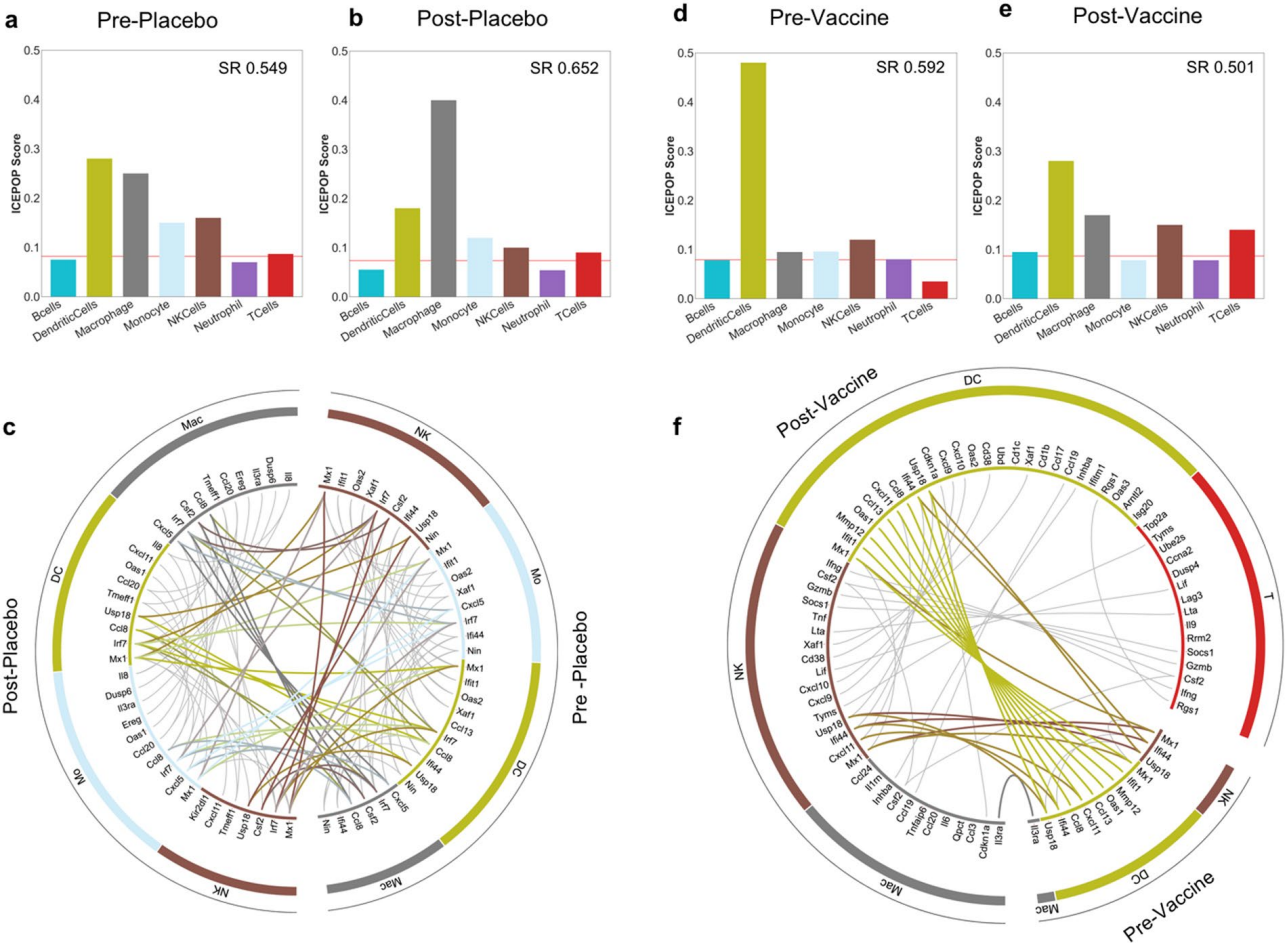
Immune cell population responses in the GSE13587 dataset. Bar plot of (**a**) pre-placebo, (**b**) post-placebo. (**c**) The circular plot of pre-placebo vs post-placebo. Bar plot of (**d**) pre-vaccine, (**e**) post-vaccine. (**f**) The circular plot of pre-vaccine vs post-vaccine. The genes are linked with coloured thick lines if they coexist in different samples; otherwise, they are linked with grey lines underneath.

ICEPOP analysis indicated dendritic cell and macrophage responses in all 4 groups (Fig. 4). The vaccine status and time point-independent responses were likely to be induced by *in vitro* HPV-VLP stimulation. HPV-VLP induces dendritic cell activation *in vitro* ^14–17^, indicating that HPV-VLP itself is immune-stimulatory, consistent with our results.

In the placebo group, there were no obvious differences between pre- and post- groups (Fig. 4a vs b). This response similarity was also demonstrated by the coloured lines in the circular map (Fig. 4c). In contrast, the same analysis of the vaccine group showed that T cells, NK cells, macrophages, and dendritic cells were more strongly activated in the post-vaccine group compared to that in the pre-vaccine group (Fig. 4d vs e, and f). Importantly, T cell responses were only observed in the post-vaccine group (Fig. 4e and f). This result suggested that HPV-VLP vaccination can successfully induced T cell responses. The listed genes of T cells and NK cells in the circular map (*Ifng, Csf2, Gzmb, Socs1, Lta, Lif, Tyms*) of the post-vaccine group (Fig. 4f) also suggested that HPV-VLP vaccination induced HPV-specific cellular immune responses^18–20^. Interestingly, gene responses in dendritic cells were also expanded in the post-vaccine (Fig. 4f). This may imply that the vaccination-dependent activation of T cells and NK cells after *in vitro* VLP stimulation also influenced the activation status of dendritic cells and macrophages in the same culture, possibly through a cytokine and/or a cell-cell contact mechanism(s) among them at the secondary level.

### ICEPOP analysis of human gut biopsy sample from inflammatory bowel disease treated with Infliximab

We applied ICEPOP to another dataset obtained from gut mucosal biopsy (GSE16879) derived from patients with inflammatory bowel disease (IBD)^21^. IBD is a chronic remitting and relapsing disease characterized by mucosal inflammation of the gastrointestinal tract. Tumour necrosis factor-alpha (TNF-α) is pivotal in the pathogenesis of IBD, therefore TNF-α blocking antibody therapy is utilized for IBD treatment^22^. The GSE16879 study involved three IBD diseases: ulcerative colitis (UC), Crohn’s ileitis (CDi), and Crohn’s colitis (CDc). All patients received TNF-α blocking monoclonal antibody infliximab (IFX). IFX responders (R) and non-responders (NR) were determined and reported previously^21^. Each patient had a biopsy before and after IFX treatment. Healthy control colon and ileum biopsy samples were included in this dataset. For calculation of DEGs, healthy colon control data was used for UC and CDc data (since they were colon biopsies), and healthy ileum control data was used for CDi data (since CDi samples were ileum biopsies) (Supplementary Fig. S1d).

**Figure 5:**
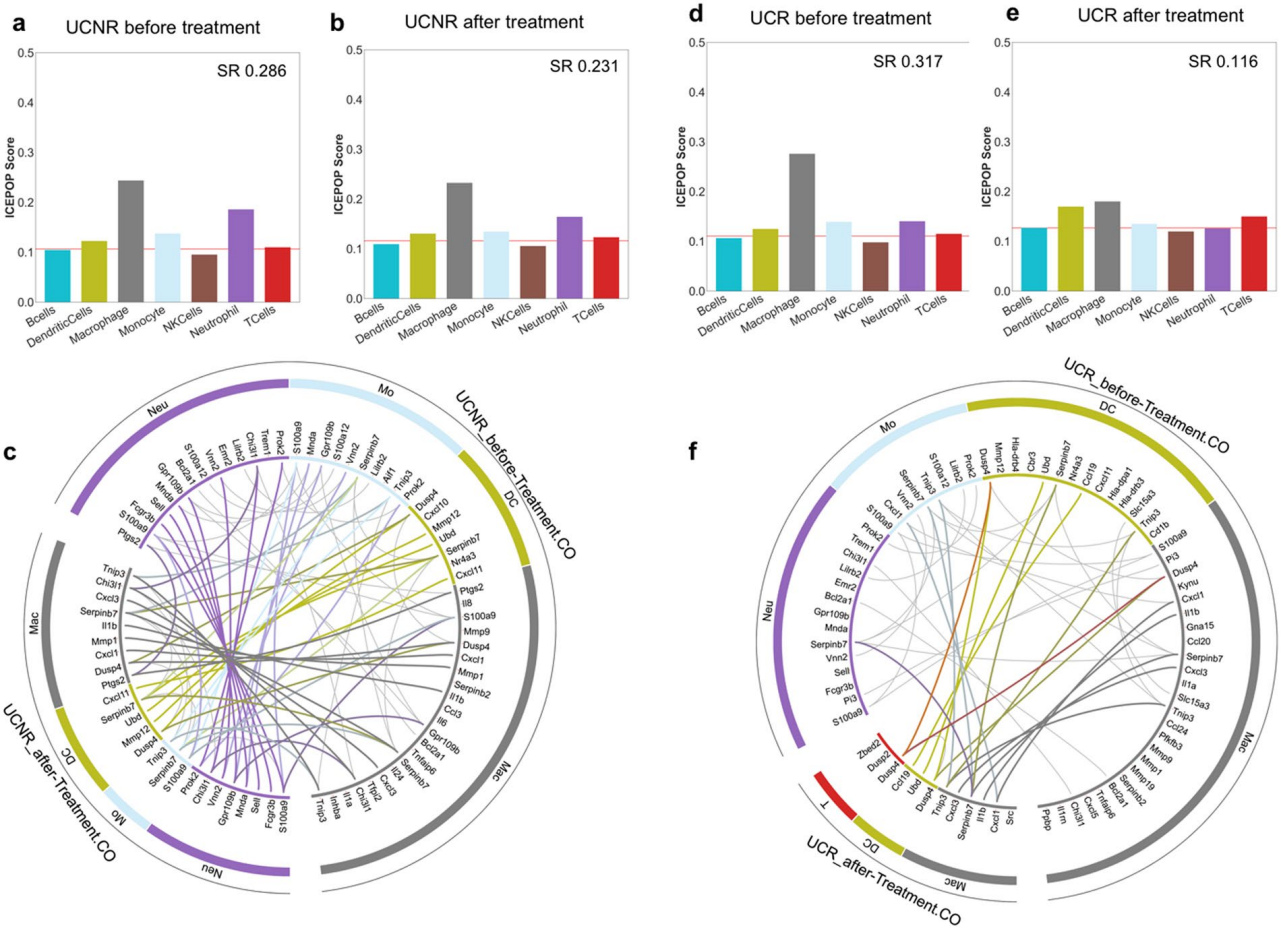
Immune cell population responses in the GSE16879 dataset. Bar plot of (**a**) UCNR before treatment, (**b**) UCNR after treatment. (**c**) The circular plot of UCNR before treatment vs UCNR after treatment. Bar plot of (**d**) UCR before treatment, (**e**) UCR after treatment. (**f**) The circular plot of UCR before treatment vs UCR after treatment. The genes are linked with coloured thick lines if they coexist in different samples; otherwise, they are linked with grey lines underneath.

In the ICEPOP analysis of UC patients, NRs showed almost identical results before and after IFX treatment (Fig. 5a vs b). This is also supported by the circular plot as many responsive genes were connected between “before” and “after”, indicating that their gene profiles were not changed with IFX treatment (Fig. 5c).

In contrast, the responses were noticeably different in UCR patients (Fig. 5d–f), where a notable decrease of macrophage activation was observed after IFX treatment (Fig. 5d vs e). A similar finding has been described^23^. The circular plot analysis further indicated that the number of listed genes was greatly reduced after treatment (Fig. 5f). Characteristically, *TNFAIP6* ^24^ was present in the pre-treatment but not post-treatment (Fig. 5f). This decrease of macrophage activation and the absence of *TNFAIP6* after IFX treatment strongly suggest the effectiveness of IFX treatment in UC responders.

Concerning CDi, ICEPOP score of neutrophil considerably decreased after IFX treatment in CDi responders (Supplementary Fig. S7d vs S7e) but not in NR (Supplementary Fig. S7a vs S7b). Previous studies suggested the involvement of neutrophil activation in CD^25,26^. The circular map analysis further indicated that the gene responses in neutrophils, monocytes, dendritic cells, and macrophages were substantially decreased after IFX treatment in the CDiR group (Supplementary Fig. S7f) but not as much in the CDiNR group (Supplementary Fig. S7c). These tendencies were further supported by an alternative DEG calculation (see Supplementary Fig. S1d), in which data obtained prior to treatment was divided by data after treatment in the same group, namely healthy controls were not used for this alternative DEG calculation. Neutrophil and monocyte responses were preferentially decreased in CDiR (Supplementary Fig. S9b) but not in CDiNR (Supplementary Fig. S9a). Of note, in this analysis, increased scores in the bar graph (Supplementary Fig. S9b) and more listed genes in circular plot (Supplementary Fig. S9c) indicated that the actual responses decreased with IFX treatment.

ICDc data revealed a similar tendency to CDi in the alternative before-after treatment analysis. In the CDcR group, the neutrophil score increased (indicating decreased neutrophil response with IFX treatment; Supplementary Fig. S9e). This was not evident in the CDcNR group (Supplementary Fig. S9d). This was similarly demonstrated by the circular plot (Supplementary Fig. S9f; increased gene list means that the actual gene responses were decreased). Interestingly, the non-alternative bar graph analysis shown in Supplementary Figure S8 (this is the same analysis for CDi group as shown in Supplementary Figure S7) provided no obvious clue of neutrophil decrease in CDcR with IFX treatment (Supplementary Fig. S8d vs S8e). Instead, circular analysis of CDc (Supplementary Fig. S8f) already suggested the decrease of neutrophil, dendritic cell, and macrophage gene responses after IFX treatment.

Taken together, these results of CD biopsy indicate that the decrease in neutrophil responses in CD patients after IFX treatment may be a general feature of the IFX responsiveness and is consistent with a recent report that neutrophil surface marker status is associated with IFX treatment effectiveness^27^. To a lesser degree, there was a small decrease in monocytes in CDiR and CDcR (Supplementary Fig. S7 and Supplementary Fig. S8), which may implicate monocyte involvement in CD pathogenesis, consistent with that suggested by recent studies^28,29^.

The collective data indicate that ICEPOP analysis can provide practical and insightful information for a variety of mouse and human derived data.

### Robustness of ICEPOP analysis

To examine the robustness of ICEPOP analysis, we first examined whether the selection of cell type affects the ICEPOP score stability. In ImmGen, 214 subtypes are categorized in 10 cell types (Supplementary Fig. S10a). Similarly, in IRIS, 22 subtypes are categorized in 7 cell types (Supplementary Fig. S10b). For this examination, we explored three different cases to make an immune cell scoring matrix. Case 1 was a matrix of all individual subtypes. Case 2 was a matrix of every cell type containing with three randomly selected subtypes of each (Supplementary Fig. S11a,b). Case 3 was a matrix of 100 randomly selected subtypes irrespective of cell type, with the number of cell type potentially being fewer than 10 depending on the selected subtypes (Supplementary Fig. S11c,d). In case 2 and 3, 1000 sampling procedures were performed for ImmGen dataset, yielding 1000 scoring matrices for each case. This permutation analysis was not performed in IRIS dataset due to the small number of available subtypes.

ICEPOP analysis using the case 1 matrix provided finer sub-cell-type responses for the same mouse and human datasets used in this study (Supplementary Fig. S10). This fine matrix may be useful when focus on very specific immune subtypes is needed. ICEPOP analysis using case 2 and 3 matrices proved nearly consistent with the default matrix as a whole (Supplementary Fig. S11), suggesting that the results of ICEPOP analysis are stable to the random subtype selections, at least for these two different permutation approaches. In the GSE6332 dataset, neutrophils in bCD.ID remained the most responsive, whereas in bCD.IP, the immune response was almost flat (Supplementary Fig. S11a and S11c). This pattern was consistent with Fig. 2. A similar tendency was also seen for GSE7768 datasets (Supplementary Fig. S11b and S11d).

We next examined the statistical significance of the ICEPOP score. For this purpose, we generated random input data to use as null distribution examples. In other words, we compared the real ICEPOP score distribution with that of random data. The fold-change value distribution was examined using the 20 real datasets (4 mouse, 16 human) complied in this study by the maximum likelihood fitting method of the R package (MASS) for each mouse and human dataset separately. This analysis revealed that the real fold-change value distribution was best fitted as a lognormal distribution (Supplementary Fig. S12) for both mouse and human data. To create the simulated null distribution data, we permuted the fold-change value 1000 times under the assumption of lognormal distribution. This yielded 1000 randomly simulated input data for ICEPOP analysis. The resultant ICEPOP score for each cell type, SR, and CRT for mouse (Supplementary Fig. S13a–c) and human (Supplementary Fig. S13d–f) was obtained. We also depicted real SR and CRT together with simulated ones. For SR score, the simulated random distribution (blue) had a very narrow spread, located to the left side of the plot (Supplementary Fig. S13b and S13e), whereas the real data (red) had a wide spread, with two peaks apparent in mice (Supplementary Fig. S13b) and several inseparable peaks in human (Supplementary Fig. S13e). The two mouse peaks corresponded to responding and non-responding real data. The null distribution overlapped with non-responding real data distribution peak in mouse (Supplementary Fig. S13b). A similar tendency was evident for the CRT score (Supplementary Fig. S13c and S13f). Random null distribution of CRT was again narrow and located at the right side of the plot. This was expected because CRT is inversely proportional to SR.

**Figure 6:**
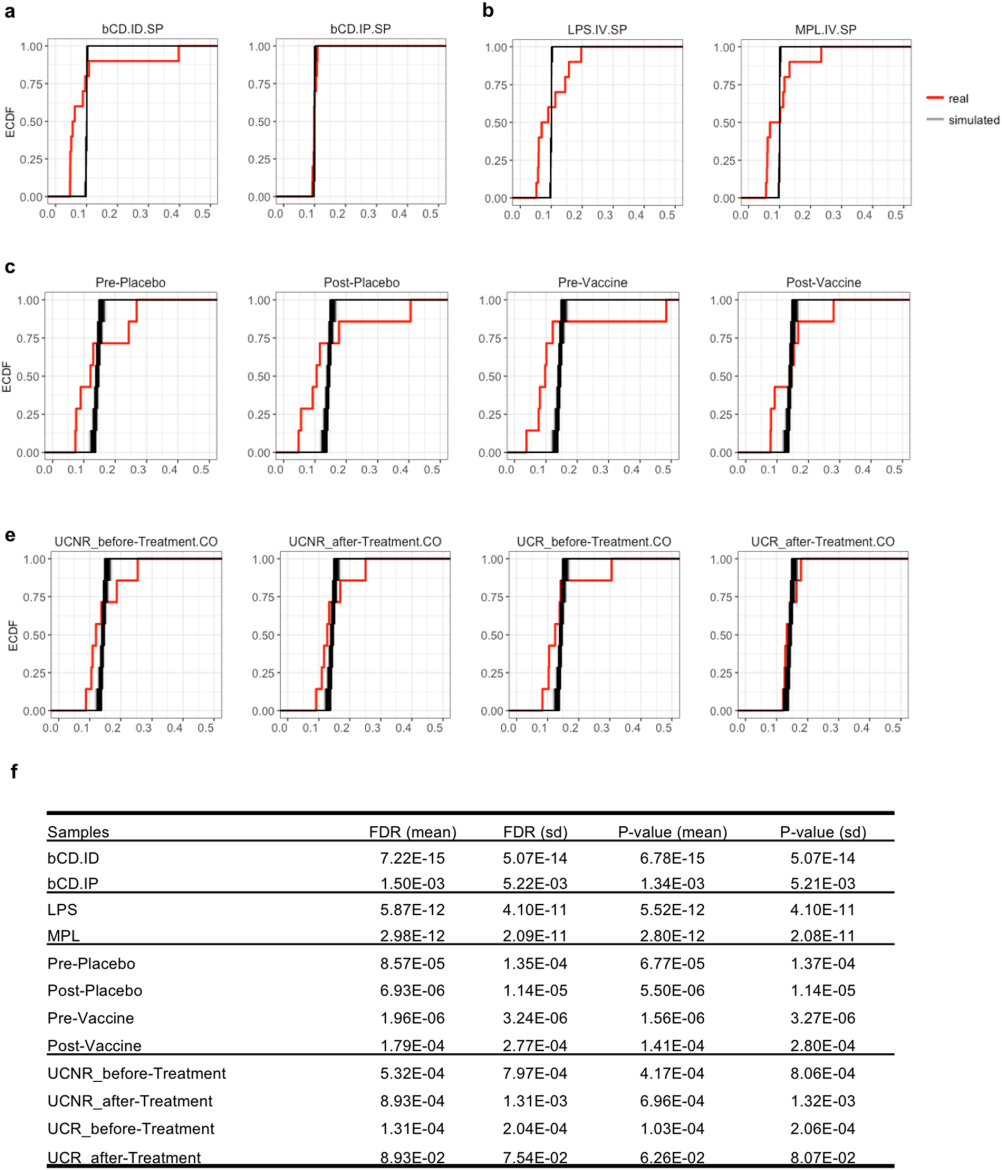
Empirical cumulative distribution function (ECDF) plot. (**a–d**) The plot juxtaposed the ICEPOP score of the real data (shown in red lines) – (**a**) GSE63332, (**b**) GSE7768, (**c**) GSE13587, and (**d**) GSE16879 – versus randomly simulated score (shown in black lines). The simulated score is generated by performing 1000 times gene permutation. (**e**) The statistical significance test of ICEPOP score with the simulated data. The p-value is determined with F-Test and FDR multiple testing adjustment.

Based on these data, we performed a statistical comparison between real and null data. ICEPOP score of the real and simulated data were depicted as an empirical cumulative distribution function (ECDF) plot (Fig. 6a–e). In these ECDF plots, the responding samples clearly separated from the random data. bCD.IP showed almost the same pattern as the null distribution (Fig. 6a), consistent with the near lack of response result of Fig. 2d. The ECDF plot of UCR_after Treatment was also nearly identical as the null plot (Fig. 6d). This case indicated that IFX treatment actually reduced pathological immune cell activation in PBMCs; therefore, their ECDF distribution was almost the same as the null ECDF plot. In HPV vaccine data, all samples were distinguished from null data (Fig. 6c). This was expected because all of these samples were stimulated with HPV-VLP *in vitro*. The statistical significance between real and null data using the F-test (FDR correction and p-value) is shown in Fig. 6e. Consistent with the ECDF plot, non-responsive samples had less significant FDR compared to the significant ones.

### Comparison with CTen and GSEA

Using the same dataset, we compared our results with the CTen^4^ web-based platform used to identify enriched cell types based on a qualitative approach using a user-provided gene list.

Supplementary Figure S14 presents the CTen analysis of all the examples used in this study. The x-axis indicates the CTen enrichment score; a cell type with a score greater than two was considered significant (http://www.influenza-x.org/~jshoemaker/CTen/). For the bCD data, the result of CTen was consistent with our estimation of the granulocytes as the highest-ranking cell type for bCD.ID (Supplementary Fig. S14a). However, the CTen results also contained relatively irrelevant terms, such as ‘Bone’ and ‘Lung’, even though the sample was derived from spleen. In the LPS analysis (Supplementary Fig. S14b), ‘Macrophage LPS’ was identified as a highly enriched term. Although the result singled out LPS terms, the majority of the highest-ranking terms reported in both LPS and MPL were apparently biased to macrophages and out-weighted granulocytes (neutrophils) (Supplementary Fig. S14b). These CTen results suggest that the gene signature enrichment approach is consistent with ICEPOP only when a sample contains a single, highly responding population (Supplementary Fig. S14a). When multiple populations respond, the CTen result is likely to be overweighed by a certain cell population, depending on the algorithm (Supplementary Fig. S14b); presently, in the LPS data, macrophage was the dominant population in CTen.

In the HPV data (GSE13587), the overall CTen scores were low, although post-vaccination showed a relatively higher score (Supplementary Fig. S14c). Nevertheless, clear cell type enrichments were not detected in HPV data. They also contain many irrelevant terms, such as ‘Tongue’ and ‘Tonsil’, for PBMC samples (Supplementary Fig. S14c).

In UC data, the overall tendency was similar to the aforementioned result (Supplementary Fig. S14d). However, we also found the consistency with our result in ‘UCR_after_Treatment’ sample, which did not show obvious responses in both CTen and ICEPOP (Supplementary Fig. S14d).

We also analysed the same dataset by GSEA using C7 immunologic gene sets (Supplementary Fig. S15). Generally, the score was low and not much different among the listed annotations. The overall results were difficult to interpret in terms of cell type responses.

Comparison among ICEPOP, CTen, and GSEA are summarised in Table 2.

**Table 2:** Comparison between Immune CEll POPulation (ICEPOP), Cell Type ENrichment (CTen), and Gene Set Enrichment Analysis (GSEA).

	ICEPOP	CTen	GSEA
Approaches	Quantitative estimation based on sorted immune cell expression database (ImmGen/IRIS)	Based on enrichment of gene list	Based on enrichment of gene list combined with gene ranking method based on KS-like statistics.
Advantages	▪ Specifies the responding cell type in a quantifiable manner▪ Specifies corresponding feature genes for each responding cell type▪ Extendable with API	▪ Wide range of cell types under different conditions▪ Uses gene symbol and Entrez Gene ID	▪ Wide range of gene sets, from various biological pathways, diseases, treatment, etc.▪ Command-line interface
Disadvantages	▪ Limited cell types▪ Limited organisms▪ Only gene symbol with fold change allowed	▪ Limited cell types▪ Limited organisms▪ Non-extendable	▪ *A priori* defined gene sets are very specific.▪ ES/NES scores tend to be unvarying.

KS: Kolmogorov–Smirnov; API: application programming interface; ES: enrichment score; NES: normalized enrichment score.

### ICEPOP web and Python package

We have made ICEPOP available as a web interface (https://vdynamics.shinyapps.io/icepop/) and Python package (https://github.com/ewijaya/icepop). The current implementation can be used to analyse data in real-time. For example, the entire analysis for Figs 2, 3, 4, and 5, took around 5 minutes to complete on a single 2.6 GHz processor. Other than cell population estimation, the package also provides methods to download data in GEO format, normalization, and hierarchical clustering class to obtain gene modules and various plotting interfaces. Thus, our method provides an efficient and practical way to quantify, visualise, and analyse immune cell responses in various studies.

## Methods

The overall framework of our procedure is illustrated in Fig. 1. ICEPOP uses DEGs (gene expression difference between control vs drug-treated) as inputs. For analysis, the sample species (mouse or human) need to be specified. If the species is mouse and if the organs are liver, spleen, and lymph node, contaminating genes from untargeted nearby organs can be filtered out by our CV reference table developed as part of the Adjuvant Database Project independent of this study. For both mouse and human samples, the DEGs for analysis need to be selected by setting the fold-change cutoff. The default cutoff was 2.0. The selected DEGs were processed for the relative activation score of each cell type defined by ImmGen or IRIS (ICEPOP score). The sections below describe these processes in more detail.

### Removing contaminating genes from nearby organs by CV filter

As shown in the Results section, contaminating genes from nearby tissue are detrimental to the analysis (Fig. 3b vs e). To remove these genes, we applied our CV filter, which was calculated using the Adjuvant Database Project reference data for each gene probe in each organ (liver, spleen, and lymph node) of the control (PBS-treated) mice (unpublished). We occasionally observed that some gene probes showed markedly variable expression values, and found that relatively high CV valued gene probes mostly derived from a non-targeted nearby organ. Preliminary experiments as part of the Adjuvant Database Project indicated that genes with CV > 1 were derived from near-by organs (Supplementary Fig. S3).

CV for each organ was calculated as follows. ${E}_{i}^{k}$ is the expression of gene *i* from mouse *k*. The variable *K* represents total number of mice, where $k\in K$. The mean and standard deviation are defined respectively by:

1 $${\bar{E}}_{i}^{K}=\frac{{\sum }_{k\in K}{E}_{i}^{k}}{|K|}$$

and

2 $${\sigma }_{i}^{K}=\sqrt{\frac{{\sum }_{k\in K}[{({E}_{i}^{K})}^{2}-{({\bar{E}}_{i}^{K})}^{2}]}{|K|-1}}$$

and the *cv* was calculated as:

3 $$c{v}_{i}=\frac{{\sigma }_{i}^{K}}{{\bar{E}}_{i}^{K}}$$

Because we do not have the equivalent reference data for human samples, and the contaminated genes are expected to be negligible in human PBMCs and human mucosal biopsies, our CV filter was not applied for HPV-VLP vaccine data (GSE13587) and UC/CD data (GSE16879).

### Immune cell scoring matrix generation from ImmGen and IRIS databases

One of the key components of our framework is a scoring matrix. To make this matrix, we utilized the ImmGen (http://www.immgen.org/)^6,30,31^ and IRIS databases (http://share.gene.com/share/clark.iris.2004/iris/iris.html)^7^. In the ImmGen data, 214 different immune cells (subtypes) were further grouped into 10 different immune cell types according to the ImmGen definition, including B cells, dendritic cells, macrophages, monocytes, NK cells, neutrophils, stem cells, stromal cells, alpha beta T cells, and gamma delta T cells (see Supplementary Fig. S10a). In the IRIS data, 22 different cell subtypes were similarly grouped into their corresponding seven cell types: including B cells, dendritic cells, macrophages, monocytes, NK cells, neutrophils, and T cells (see Supplementary Fig. S10b).

The *Immune Cell Scoring Matrix* (Supplementary Fig. S2c) was generated as shown in Supplementary Figure S1a–c. In this process, we denoted a gene *i*, $i\in G$; it is the index of a gene in the set *G (21,755 genes in ImmGen and 1,654 genes in IRIS)*. The gene expressions are annotated as ${E}_{i}^{j}$,$j\in C$; *j* is the index of the individual 214 or 22 different subtypes within grouped immune cell type *C*. *C* is the index of 10 or 7 different cell types defined in ImmGen or IRIS, respectively. For example, 26 subtypes including B.T1.Sp and B.Fo.Sp were grouped under B cells (see Supplementary Fig. S10a). The averaged gene expression values for *C* (10 or 7 different cell types) were calculated (see Supplementary Fig. S2a) as:

4 $${w}_{i}^{C}=\frac{{E}_{i}^{j}}{{\sum }_{j\in C}{E}_{i}^{j}}$$

These values were further normalized for gene-probes (row-wise) (Supplementary Fig. S2b) by the following equation:

5 $${\hat{w}}_{i}^{C}=\frac{{w}_{i}^{C}}{{\sum }_{\forall C}{w}_{i}^{C}}$$

We then normalized for cell types (column-wise) (Supplementary Fig. S2c) by the following equation:

6 $${\widehat{\hat{w}}}_{i}^{C}=\frac{{\hat{w}}_{i}^{C}}{{\sum }_{i\in G}{\hat{w}}_{i}^{C}}$$

The resultant is the *Immune Cell Scoring Matrix*.

### Quantitative estimation of immune cell type responses

DEGs between control (such as PBS) and drug-treated (such as LPS-injected and IFX-treated) were calculated as fold-changes. We focused on upregulated genes and selected DEGs based on a fold-change >2.0 (Supplementary Fig. S2d). The selected DEG fold-change values were used as a multiplying factor for the *Immune Cell Scoring Matrix* in equation (6), where ${F}_{i}$ is the fold-change of gene *i*. The factor ${F}_{i}{\widehat{\hat{w}}}_{i}^{C}$ was termed the *gene level response score* (Supplementary Fig. S2e; it shows gene level response table). It represents the degree of contribution of a particular gene to the *ICEPOP score*.

The final estimated response of cell type *C* was calculated by aggregating and normalizing the contribution for all cell types. It is expressed as:

7 $$Rc=\frac{{\sum }_{i\in G}{F}_{i}{\widehat{\hat{w}}}_{i}^{C}}{{\sum }_{i\in G,\forall C}{F}_{i}{\widehat{\hat{w}}}_{i}^{C}}$$

The outcome is the *immune cell response score* (*R*_*c*_) (Supplementary Fig. S2f). Of note, *R*_*c*_ is calculated from the pooled samples where both treated and control samples are already averaged from the replicates. In our subsequent descriptions, we use the phrase *ICEPOP score* to denote *R*_*c*_.

### CRT and SR score

We also defined the cell type response threshold (CRT). If a cell type has an *ICEPOP score* over this threshold, the cell type is considered as activated or responsive to the treatment. When *R* is a set of *ICEPOP scores* of all the cell types (such as 10 cell types in ImmGen and 7 cell types in IRIS based analysis), then the CRT is the lower quartile of the *R* (*R*_*q1*_), which is the median of the lower half of *R*. As a caution, CRT is a relative threshold, therefore it depends on the number of the cell types (*C*_*total*_). CRT theoretically ranges from 0 to 1/*C*_*total*_. For example, in the case of hypothetical data with constant DEGs (Supplementary Fig. S2h) of 5 cell types, the threshold will be 0.2 (i.e. one-fifth) (Supplementary Fig. S2j and S2k), which is the highest CRT score in this constant example and indicates that this exampled sample showed no responses. Similarly, the theoretical highest CRT for ImmGen is 0.1 (one-tenth) and for IRIS is 0.143 (one-seventh). CRT is shown as a red horizontal line in the bar graph.

Using *C*_*total*_, we further calculated the *sample response score* (SR). It was defined as:

8 $$SR=-\mathrm{ln}({R}_{q1}\cdot {C}_{total})$$

This value represents the activation status of the whole sample (such as spleen, PBMC, or the whole biopsy tissue). When the SR score is zero, the sample was considered to have no differentially responding cells (Supplementary Fig. S2j). As equation (8) shows, when CRT = *R*_*q1*_ decreases, the SR increases. Thus, theoretically when the CRT reaches zero, the SR approaches infinity. However, in practice (for example, when CRT is 0.05), the SR is around 1.0 by ImmGen based analysis. The example in Fig. 2a shows the SR is 0.723, indicating this sample has a strongly responding cell type to the treatment. Similarly, in the case of IRIS analysis, when the CRT is 0.05, the SR is around 1.03 (Supplementary Fig. S7a). For the non-responding case (Fig. 2b) when the CRT approximate 1/*C*_*total*_ (0.1), the SR is reduced to 0.025. Although it would not occur in the real case, when all the cell types equally responded to the stimulation (as shown in Supplementary Fig. S2h), our method labelled that the sample did not respond to the stimulation. We cautioned that our method identified the sample responses depending on the differential responses among the cell types.

### Gene probe level comparison analysis with circular map presentation

The circular maps were generated using Circos^32^. The genes presented were obtained by combining the *gene level response scores* from two samples into one list table, ranking the genes by the score, and selecting the top 100 genes (Supplementary Fig. S2d,e,l–n). Genes that co-occurred in different samples are linked with coloured lines, and if they appeared within the same samples, they were coloured with grey lines. This circular map presentation visually highlights the differences of two comparing samples.

### Statistical testing

We examined the significance of ICEPOP scoring with statistical testing. We created null input data by randomly generating the fold-change values assuming a lognormal distribution, which was the best fit model for the real datasets used in this manuscript (4 from mouse and 16 from human) using the R MASS package. We then permuted 1000 times, and calculated the simulated ICEPOP score (Supplementary Fig. S13) followed by the statistical significance between the real and simulated data by F-test.

### Histology

C57BL/6 male, 6-week-old mice were administered ID (tail base) or IP with 30% HP-β-CD in PBS (total volume per mouse, 200 μl). After 6 hours, mice were sacrificed, spleens were removed, and cryosections were prepared as described previously^33,34^. All the animal experiments were approved and performed in accordance with the guidelines of the Animal Care and Use Committee of the Research Institute for Microbial Diseases, Osaka University (Osaka, Japan). The cryosections (6 μm-thick) were fixed with 4% paraformaldehyde in PBS (pH 7.4) for 5 minutes and blocked with StartingBlock Blocking Buffer (Thermo Fisher Scientific) for 10 minutes. They were then incubated with fluorescent-dye-conjugated phalloidin and antibodies (Alexa Fluor 350 Phalloidin; Thermo Fisher Scientific, Alexa Fluor® 647 anti-mouse Ly-6G Antibody 1A8; BioLegend, and PE anti-mouse/human CD45R/B220 Antibody RA-6B2; BioLegend). Fluorescent images were obtained using an Olympus IX83 inverted microscope with a DP80 digital camera and 10x objective lens (UPlanFL N, NA = 0.30, Olympus). All images were acquired and processed by cellSens Dimension software (Olympus).

## Electronic supplementary material

Supplementary information
