# Supplementary material for: Quantifying the relative immune cell activation from whole tissue/organ-derived differentially expressed gene data
Source: Sci Rep. 2017 Oct 9;7:12847. doi: 10.1038/s41598-017-12970-8 (PMC5634445; doi:10.1038/s41598-017-12970-8)
Supplement: Supplementary file 1 — Supplementary information [file 41598_2017_12970_MOESM1_ESM.pdf]

Supplementary information

Quantifying the relative immune cell activation from whole tissue/organ-derived differentially expressed gene data

Edward Wijaya[1,2], Yoshinobu Igarashi[3], Noriyuki Nakatsu[3], Yasunari Haseda[7], Joel Billaud[1], Yi-An Chen[4], Kenji Mizuguchi[4], Hiroshi Yamada[3], Ken Ishii[5,6], and Taiki Aoshi[7]*

[1] System Immunology Laboratory, Immunology Frontier Research Centre, Osaka University, Osaka
565-0781, Japan

[2] Department of Genome Informatics, Research Institute for Microbial Diseases, Osaka University, Osaka 565-0781, Japan

[3] Toxicogenomics-Informatics Project, National Institute of Biomedical Innovation, Health and Nutrition, Osaka 567-0085, Japan

[4] Bioinformatics Projects, National Institute of Biomedical Innovation, Health and Nutrition, Osaka 567-0085, Japan

[5] Vaccine Science Laboratory, Immunology Frontier Research Centre, Osaka University, Osaka 565-0781, Japan

[6] Laboratory of Adjuvant Innovation, National Institute of Biomedical Innovation, Health and Nutrition, Osaka 567-0085, Japan

[7] Vaccine Dynamics Project, BIKEN Innovative Vaccine Research Alliance Laboratories, Osaka University, Osaka 565-0871, Japan

* To whom correspondence should be addressed. Tel: +81-6-6877-4789; Fax: +81-06-6877-4790; E-mail: aoshi@biken.osaka-u.ac.jp

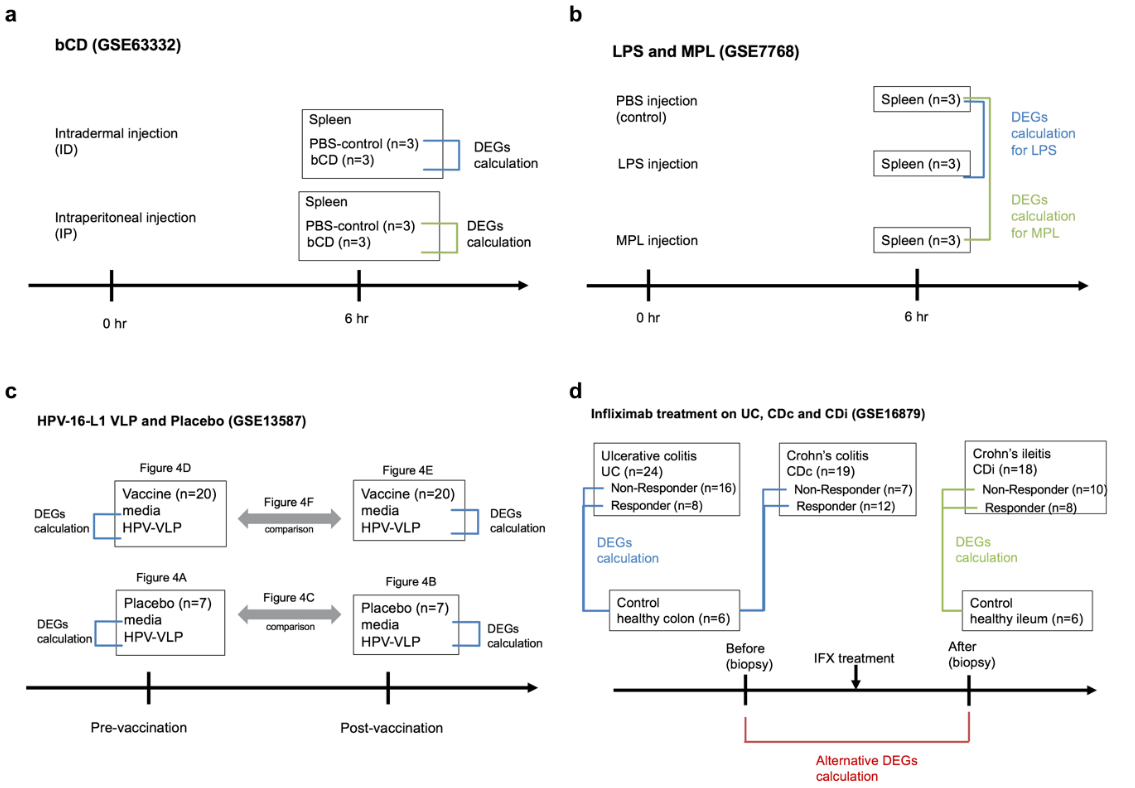

Supplementary Figure S1. Schematic diagram of the experiment and DEG calculations.

(a) bCD study in mice (GSE63332), (b) LPS and MPL study in mice (GSE7768), (c) HPV vaccine study in human PBMC (GSE13587), and (d) IBD treatment study in human gut biopsy (GSE16879).

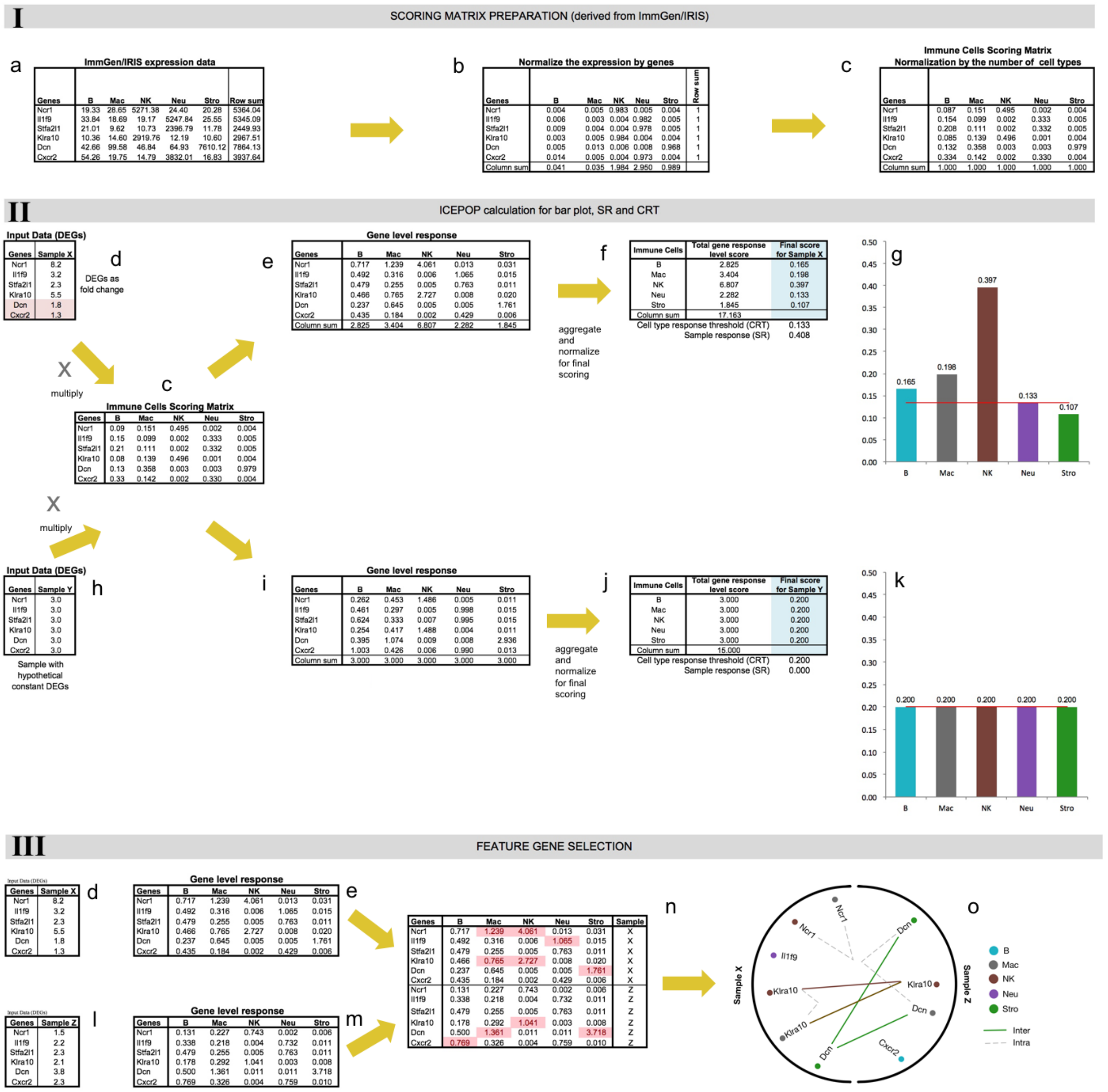

Supplementary Figure S2. Series of steps used to calculate the ICEPOP score.

I) Generation of the Immune Cell Scoring Matrix. II) Processing for calculation of ICEPOP score, cell type response threshold (CRT), and sample response score (SR). The CRT indicates the limit where a cell type is considered activated. SR is a general indicator of overall sample activation; when SR is zero, the sample has no responding cells. For simplicity, we show an example with 5 cell types instead of 10 cell types used in our main manuscript. III) The feature gene selection process. The gene level response score is calculated from two input samples—X and Z. The top K highest scoring genes (marked with red) from the X and Z combined table (n) are selected. In this example, we use K=10 for simplicity. In the main manuscript, we use K=100. Finally, those feature genes and cell types are presented in a circular plot, in which genes are connected with thick lines if they come from different samples and with thin dashed lines if they come from the same sample. These steps (I, II, III) correspond to the description in the Methods.

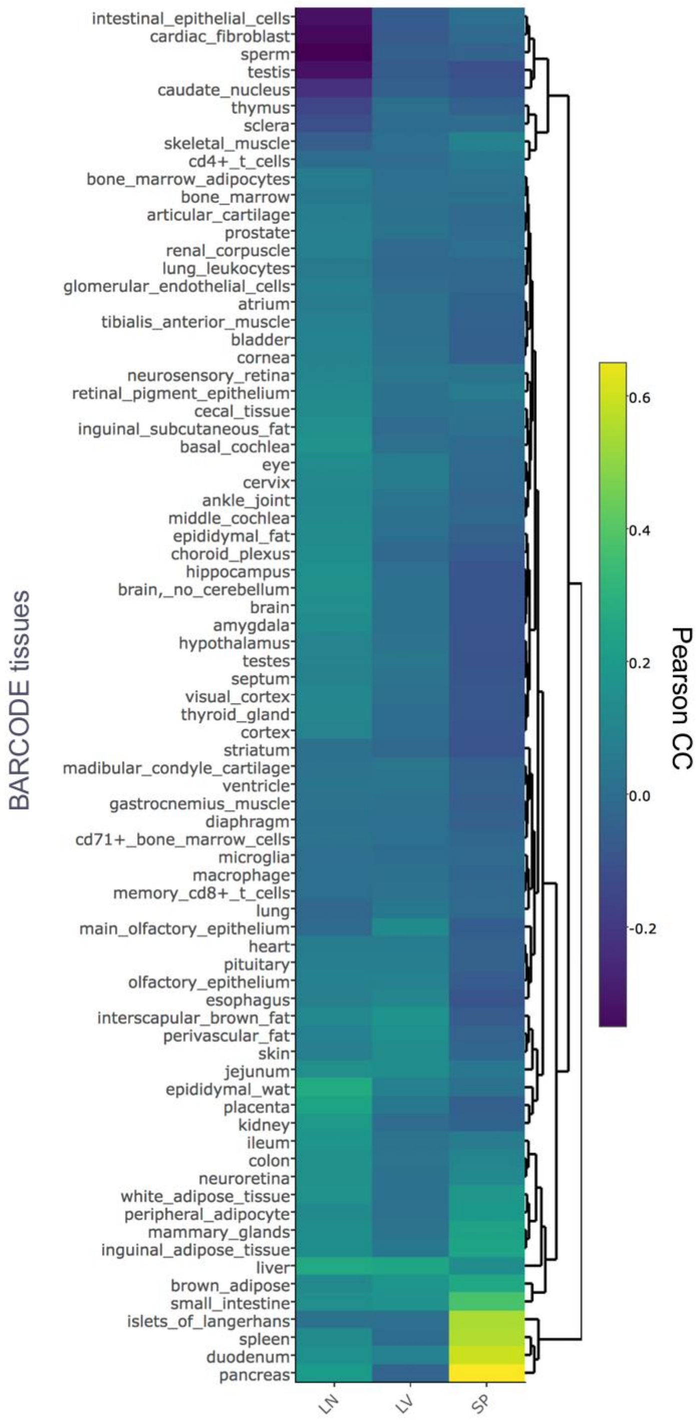

Supplementary Figure S3. Pearson correlation heat map between the coefficient of variation (CV) reference score and BARCODE 3.0 entropy scores.

The PBS control reference microarray data from adjuvant database project of CV greater than one were used. CV > 1.0 genes in control spleens were likely to be derived from the pancreas and duodenum. LN: lymph node, LV: liver, SP: spleen.

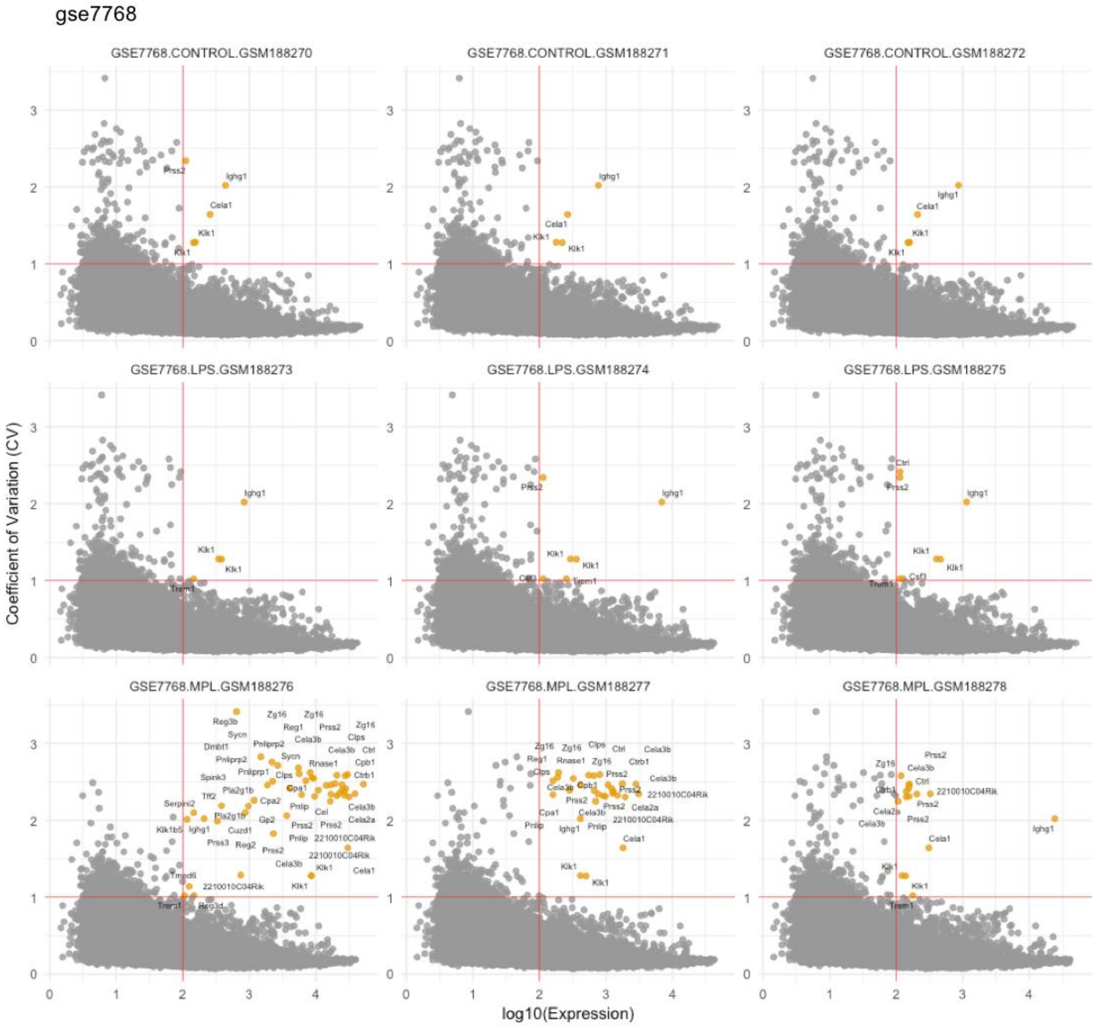

Supplementary Figure S4. Scatter plot of GSE7768 dataset (LPS and MPL study in mice). The x-axis corresponds to the gene expression and y-axis corresponds to the coefficient of variation (CV) of the adjuvant database reference. Each column refers to replicates of samples (control, LPS, and MPL). The third row shows that MPL administered spleen samples containing the contaminated genes, which are highly expressed (fold change > 100) and have a high CV (>1.0).

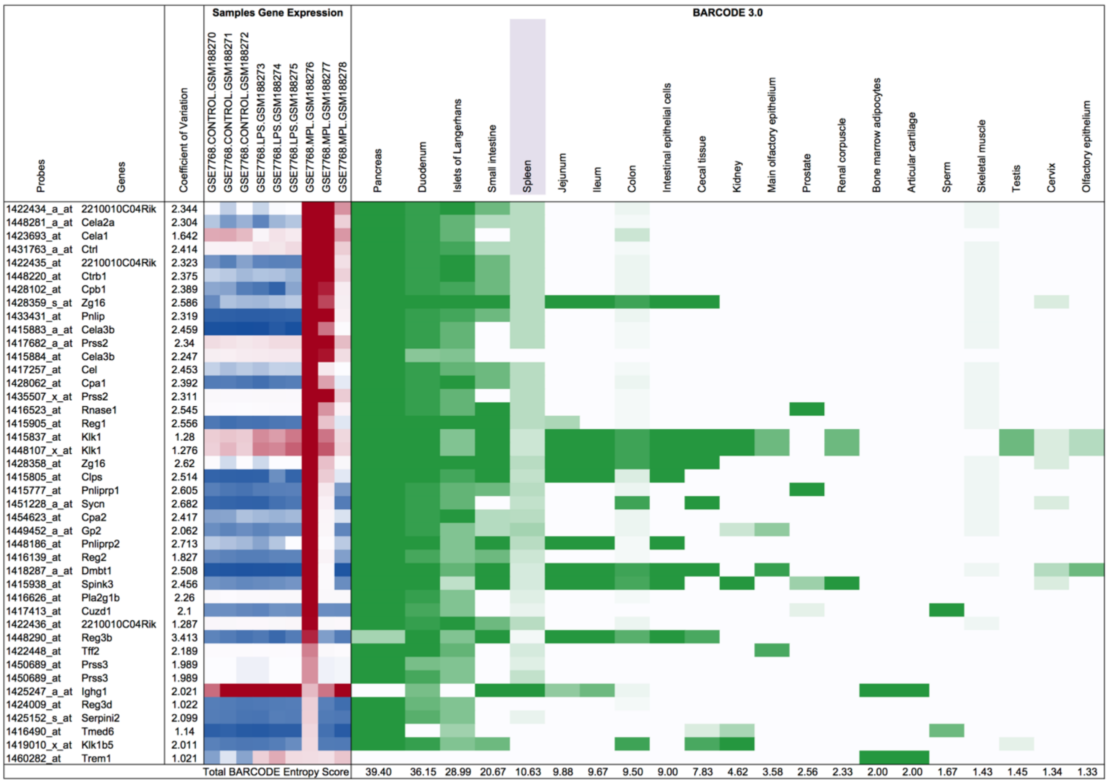

Supplementary Figure S5. A heat map showing the correlation contaminated genes in GSE7768 and BARCODE 3.0 organ entropy scores.

We identified 43 genes with an expression value > 100 and a CV > 1.0 as in Supplementary Figure S4. The left six samples are considered as non-contaminated. On the other hand, MPL spleen samples are mostly contaminated. Most of the 43 genes clearly corresponded to the pancreas and intestine by BARCODE 3.0. This suggests that MPL spleen samples were contaminated with the genes of “spleen nearby organs” including pancreas and duodenum.

a

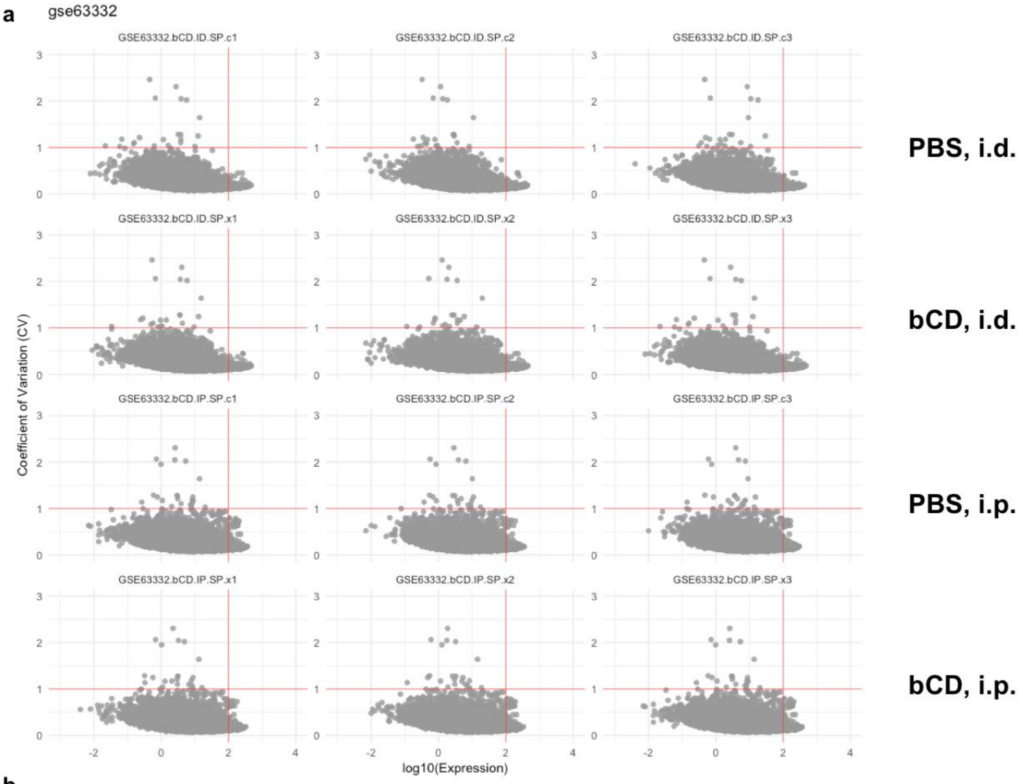

b

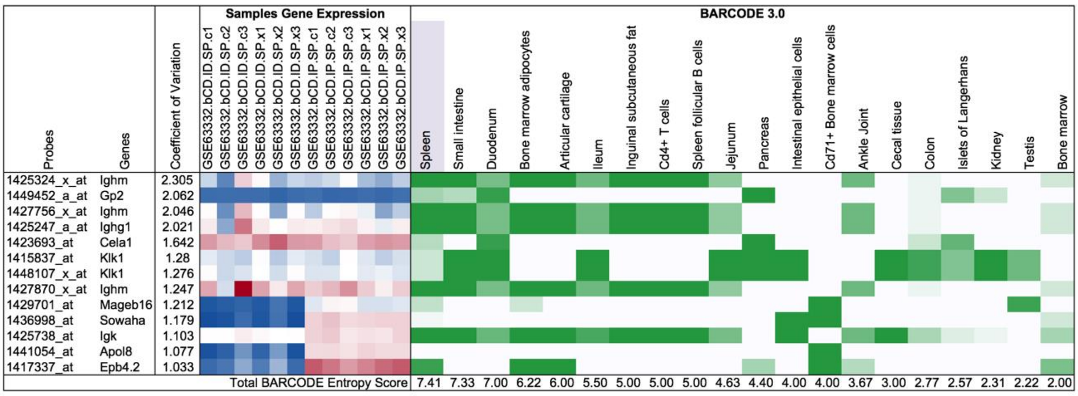

Supplementary Figure S6. Scatter plot and heat map of GSE63332 dataset.

(a) Scatter plot of GSE63332 dataset (bCD study in mice) as shown in Supplementary Figure S4. Each column refers to replicates of samples (control ID, bCD.ID, control IP, and bCD.IP). Based on our criteria there are no contaminated genes in these samples. (b) A heat map of gene with CV > 1.0 in GSE63332 as in Supplementary Figure S5. We identified 13 genes with CV > 1.0 (no expression criteria applied). In these genes, the highest correlated tissue was “Spleen” (with BARCODE Entropy score 7.41), which was the tissue from which the samples were originally derived, indicating that these samples were not contaminated with nearby organs.

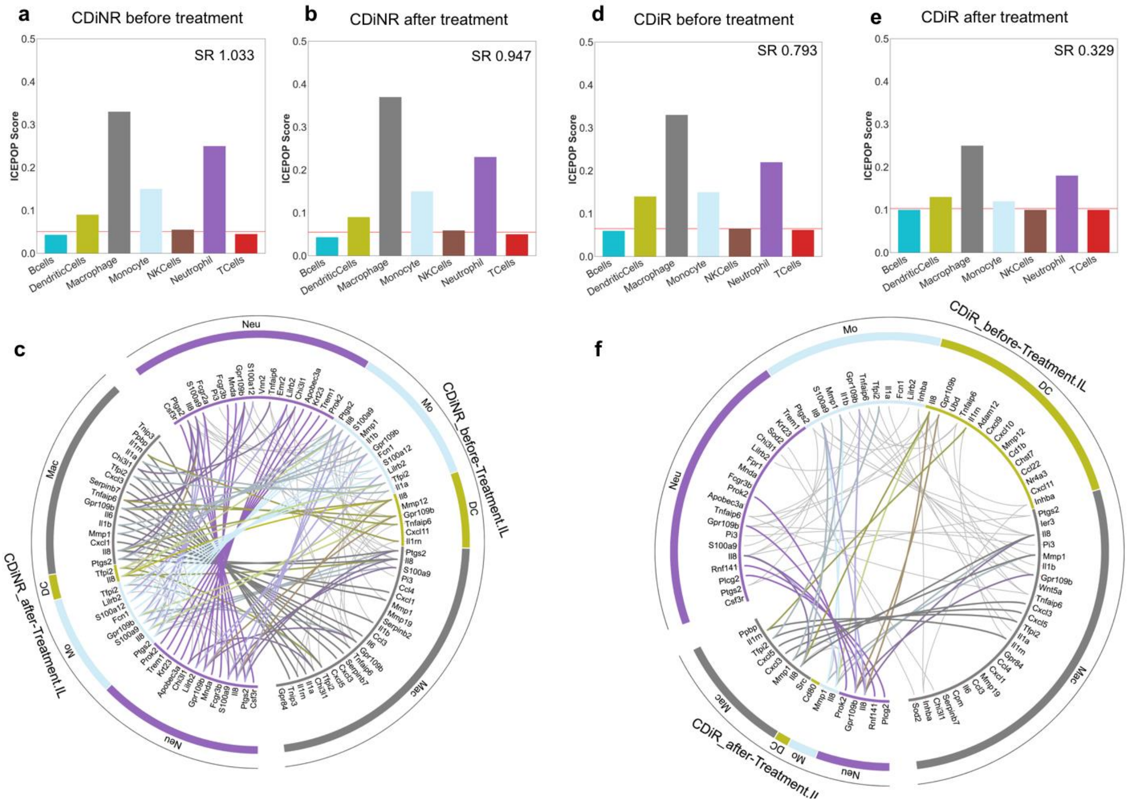

Supplementary Figure S7. ICEPOP analysis of Crohn's disease ileitis in the GSE16879 dataset.

(a and b) Crohn's Disease ileitis Non-Responder (CDiNR) (a) before and (b) after infliximab (IFX) treatment, and (c) their circular plot. (d and e) Crohn's Disease ileitis Responder (CDiR), (d) before and (e) after IFX treatment, and (f) their circular plot. The control samples used for this analysis were healthy ileum (see Supplementary Figure S1d).

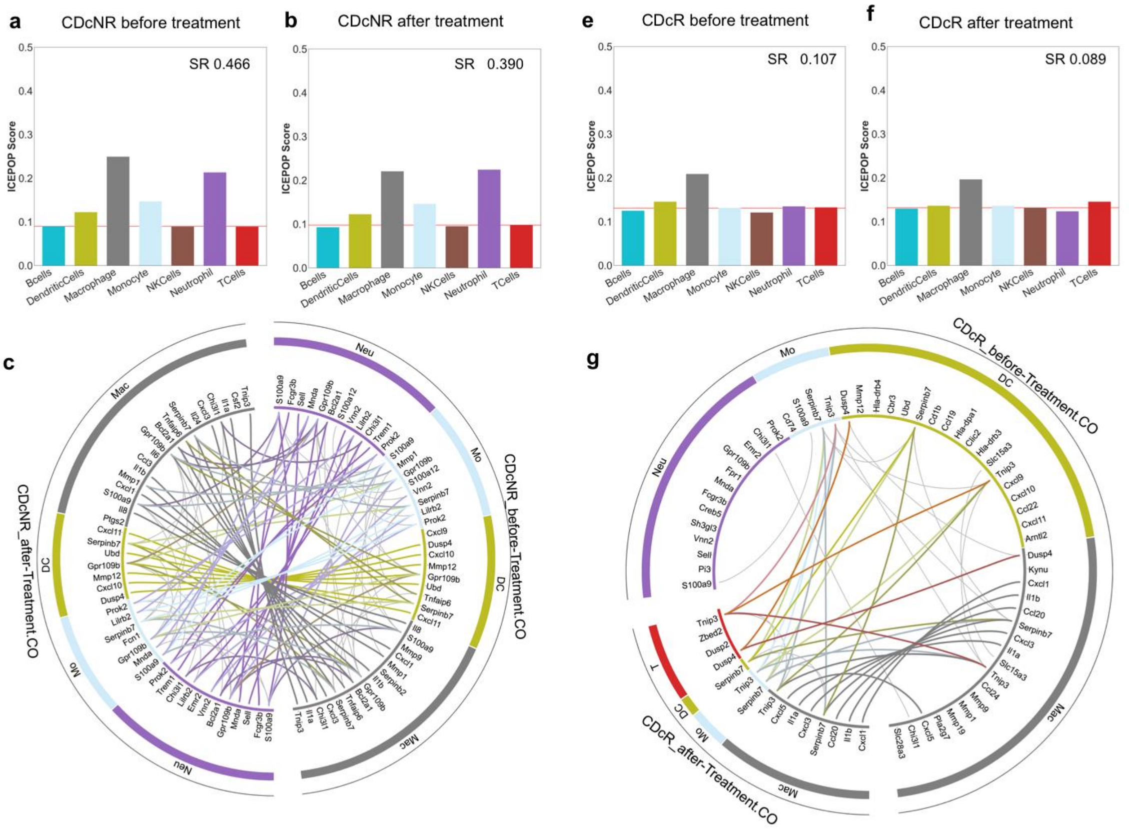

Supplementary Figure S8. ICEPOP analysis of Crohn’s disease Colitis in the GSE16879 dataset.

(a and b) Crohn’s Disease Colitis Non-Responder (CDcNR), (a) before and (b) after IFX treatment, and (c) their circular plot. (d and e) Crohn’s Disease Colitis Responder (CDcR), (d) before and (e) after IFX treatment, and (f) their circular plot. The control samples used for this analysis were healthy colon tissue (see Supplementary Figure S1d).

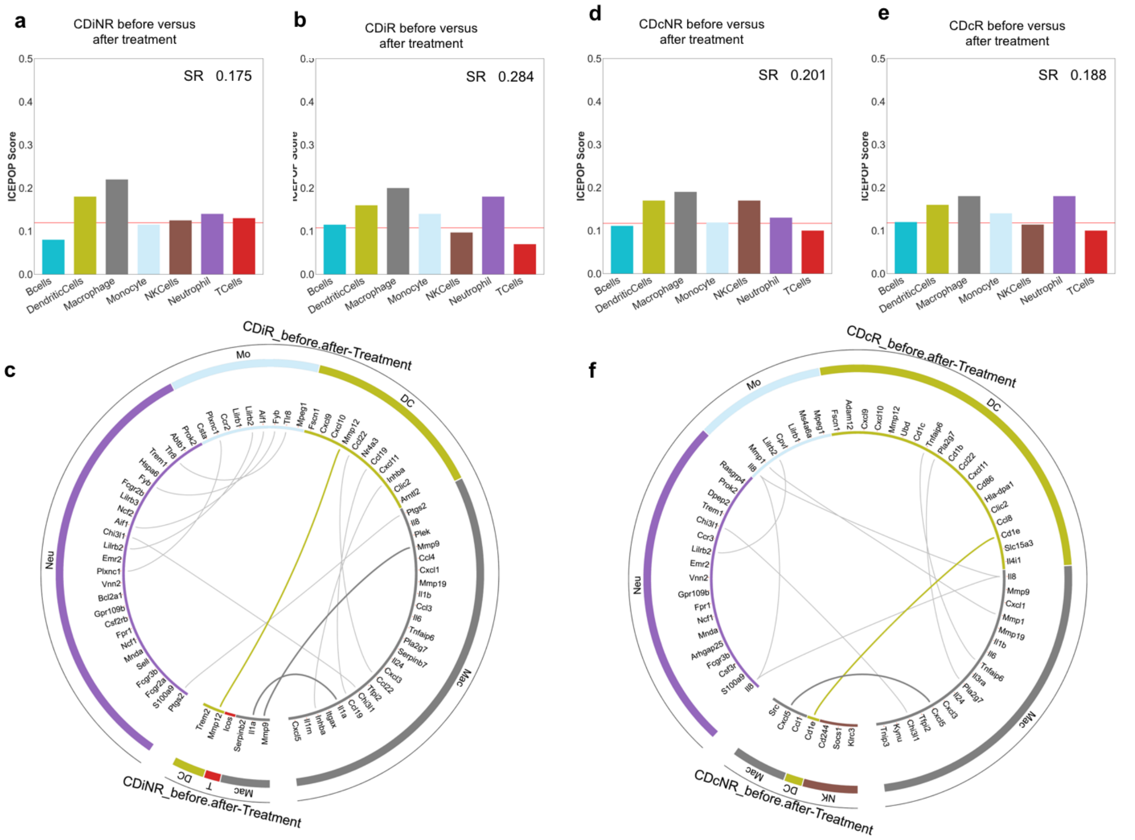

Supplementary Figure S9. ICEPOP analysis of Crohn's disease ileitis and colitis in GSE16879 dataset by using alternative DEGs as input (see Supplementary Figure S1d). (a) CDiNR before versus after treatment, (b) CDiR before versus after treatment, (c) circular plot of (a) vs (b), (d) CDcNR before versus after treatment, (e) CDcR before versus after treatment, and (f) circular plot of (d) vs (e). Of note, in this alternative DEG analysis, increased scores indicate that the actual cell responses were decreased after IFX treatment.

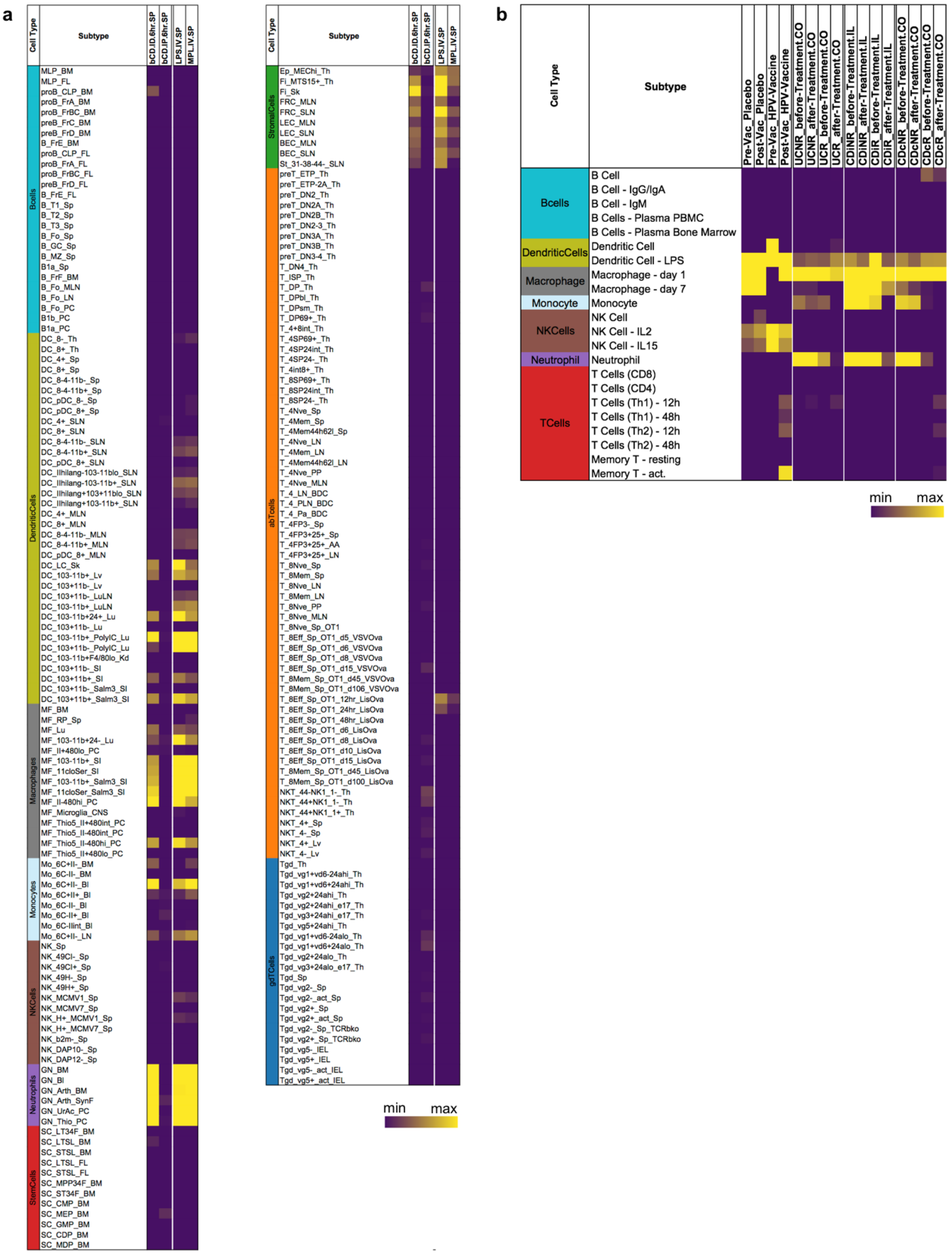

Supplementary Figure S10. ICEPOP analysis for all subcell types in ImmGen and IRIS. Heatmap of immune cell response in (a) GSE63332 and (b) GSE7768 datasets calculated using 214 ImmGen and 22 IRIS individual subcell types, instead of 10 and 7 cell types, respectively.

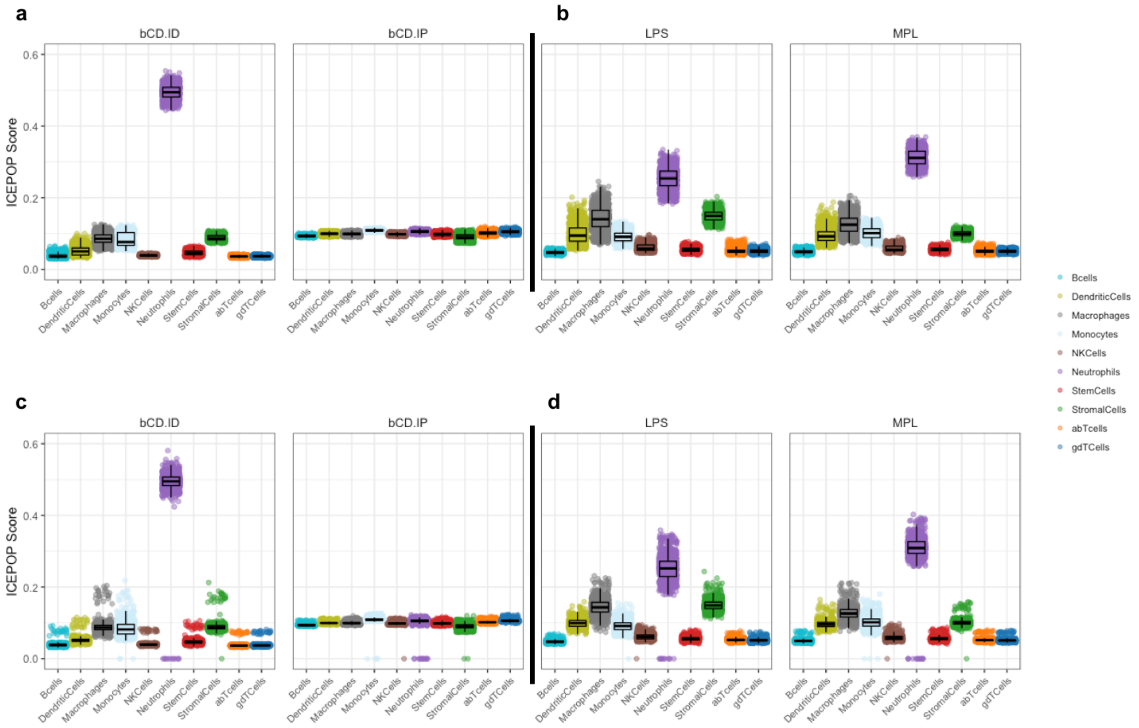

Supplementary Figure S11. Stability test of scoring matrix by random subtype selections. ICEPOP score calculation for (a, c) GSE6332 and (b, d) GSE7768 datasets. (a and b) Calculation involving 1000 random samplings of three subtypes for every parent cell type (always 10), for a total of 30 subtypes. (c and d) Calculation involving 1000 random samplings of 100 subtypes of 214 ImmGen cell types (not necessarily containing all 10 cell types, but always containing 100 cell types for each sampling permutation). In this case, the matrix may miss some of the cell types, which corresponds to the 0 score dots in the graphs (c and d).

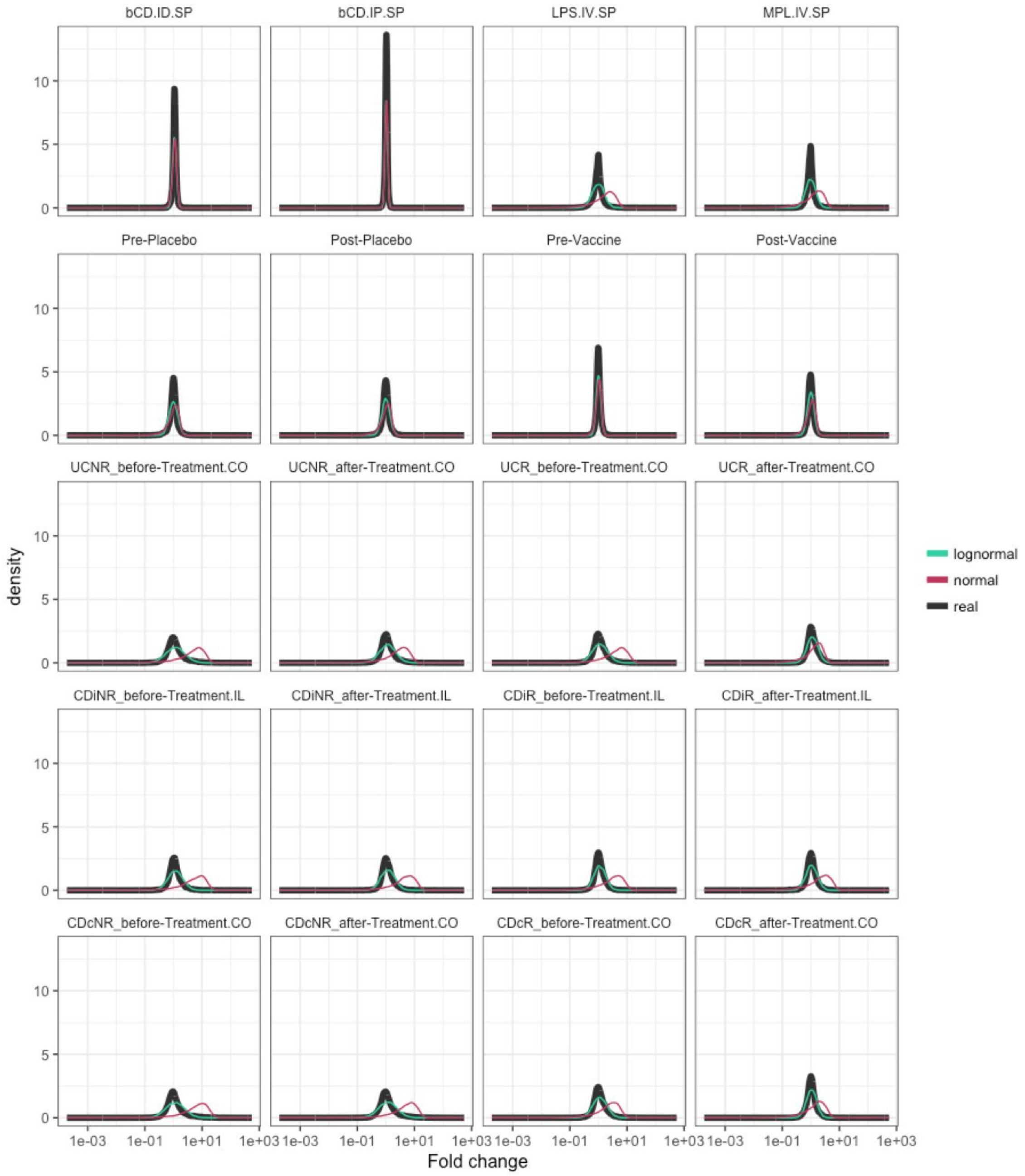

Supplementary Figure S12. Fold-change distribution from the real data used in this manuscript.

The distribution was compared with theoretical lognormal and normal distribution. The real fold-change was distributed in a lognormal pattern.

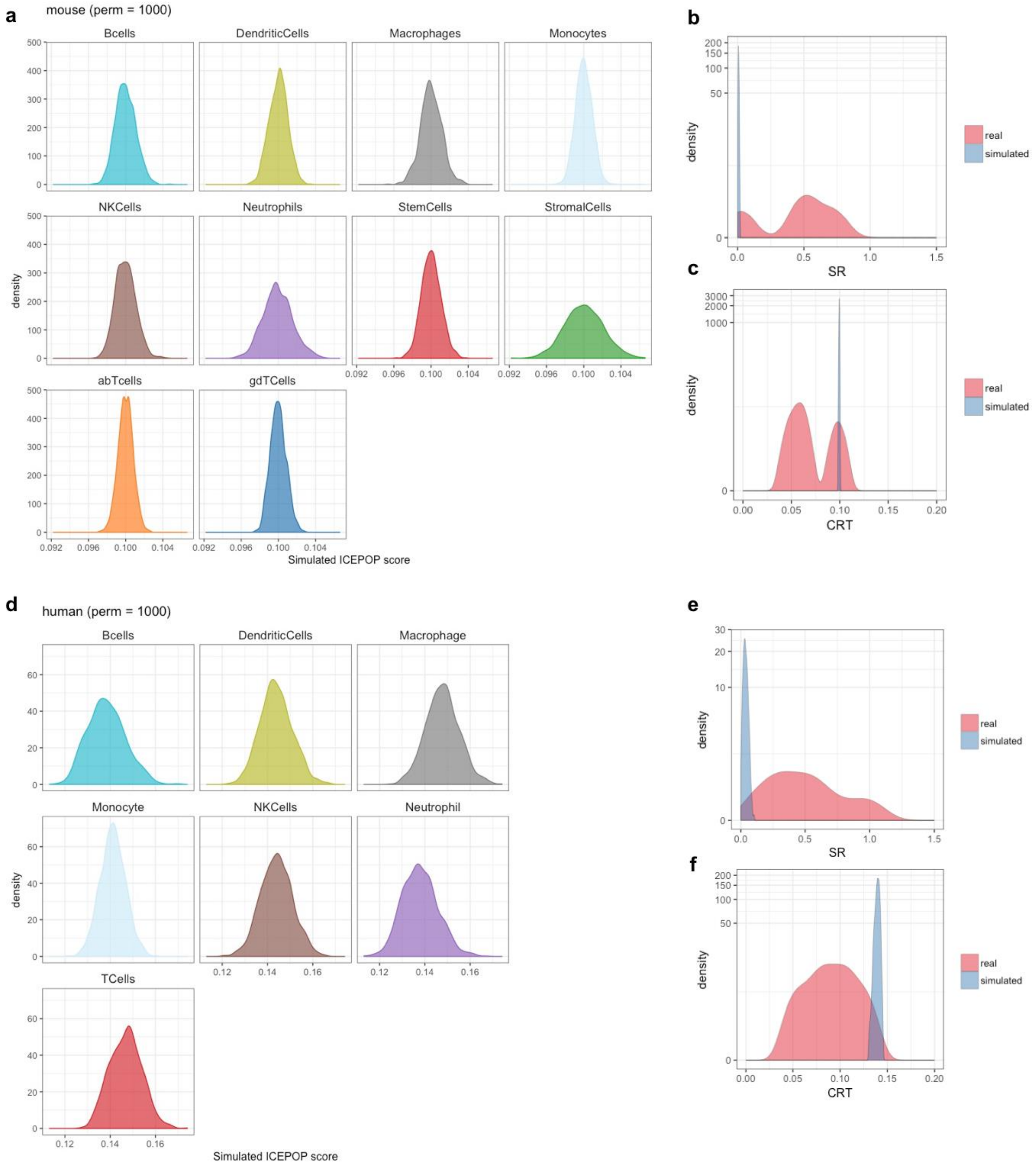

Supplementary Figure S13. Simulated null distribution of ICEPOP socre.

Simulated fold-change data was prepared for mouse (a-c) and human (d-f) by 1000 permutations based on the lognormal distribution as examined in Supplementary Figure S12. (a,d) Null distribution of ICEPOP score, (b,e) sample response score (SR), and (c,f) cell type response threshold (CRT) are shown. Real data distribution is also shown in red for (b-c) mouse and (e-f) human.

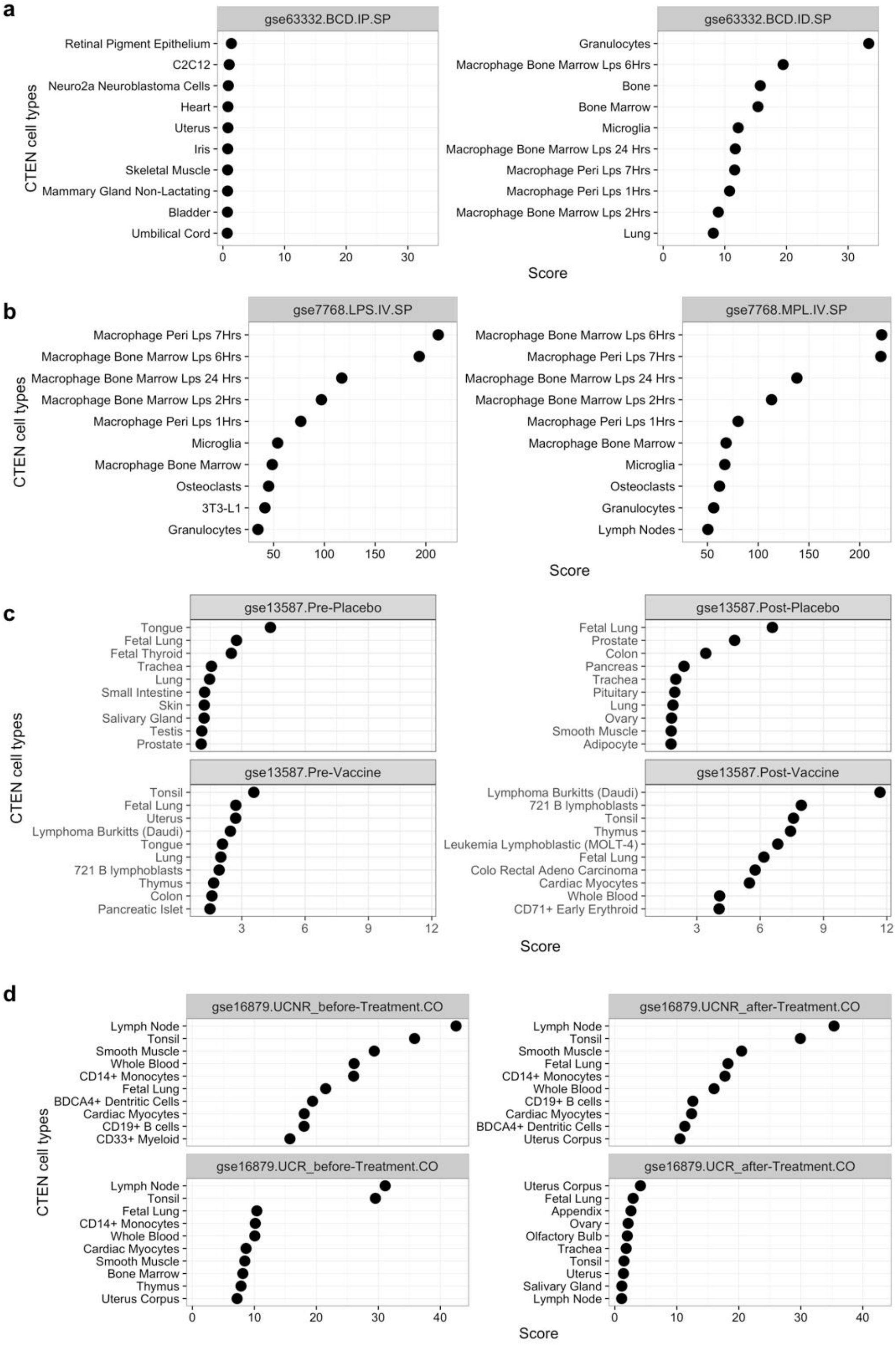

Supplementary Figure S14. CTen enrichment scores.

Only the top ten highest scoring terms are shown from the (a) GSE6332 (bCD) dataset, (b) GSE7768 (LPS/MPL), (c) GSE13587 (HPV), and (d) ulcerative colitis (UC) in GSE16879 datasets. Data used for CTen analysis are the same as those used for ICEPOP analysis.

Sample	GSEA Gene Sets	ES	NES	NOM p-val	FDR q-val	FWER p-val
LPS.IV.SP	GSE17721_LPS_VS_CPG_24H_BMDM_UP	0.510	1.884	0.000	0.140	0.060
	GSE22196_HEALTHY_VS_OBESE_MOUSE_SKIN_GAMMADELTA_TCELL_UP	0.519	1.794	0.000	0.110	0.140
	GSE13485_CTRL_VS_DAY3_YF17D_VACCINE_PBMC_UP	0.499	1.791	0.000	0.100	0.140
	GSE6674_UNSTIM_VS_ANTI_IGM_STIM_BCELL_DN	0.601	1.790	0.000	0.095	0.140
	GSE1112_HY_CD8AB_VS_HY_CD8AA_THYMOCYTE_RTOC_CULTURE_DN	0.527	1.779	0.000	0.092	0.140
	GSE43863_NAIVE_VS_TFH_CD4_EFF_TCELL_D6_LCMV_DN	0.640	1.774	0.000	0.090	0.140
	GSE17721_CPG_VS_GARDIQUIMOD_24H_BMDM_UP	0.414	1.772	0.000	0.089	0.140
	GSE1460_DP_THYMOCYTE_VS_NAIVE_CD4_TCELL_CORD_BLOOD_DN	0.414	1.748	0.000	0.088	0.140
	GSE40666_WT_VS_STAT4_KO_CD8_TCELL_UP	0.517	1.747	0.000	0.087	0.140
	GSE36392_TYPE_2_MYELOID_VS_MAC_IL25_TREATED_LUNG_UP	0.481	1.719	0.000	0.086	0.140
MPL.IV.SP	GSE6674_UNSTIM_VS_ANTI_IGM_STIM_BCELL_DN	0.613	1.845	0.000	0.100	0.000
	GSE43955_TH0_VS_TGFB_IL6_TH17_ACT_CD4_TCELL_10H_DN	0.468	1.796	0.000	0.109	0.120
	GSE17721_LPS_VS_CPG_24H_BMDM_UP	0.520	1.754	0.000	0.118	0.140
	GSE17721_LPS_VS_GARDIQUIMOD_24H_BMDM_UP	0.573	1.751	0.000	0.113	0.140
	GSE22611_MUTANT_NOD2_VS_CTRL_TRANSDUCED_HEK293T_CELL_UP	0.439	1.741	0.000	0.114	0.140
	GSE21546_UNSTIM_VS_ANTI_CD3_STIM_SAP1A_KO_AND_ELK1_KO_DP_THYMOCYTES_UP	0.755	1.736	0.000	0.112	0.140
	GSE36891_UNSTIM_VS_POLYIC_TLR3_STIM_PERITONEAL_MACROPHAGE_UP	0.678	1.731	0.000	0.113	0.140
	GSE36392_TYPE_2_MYELOID_VS_MAC_IL25_TREATED_LUNG_UP	0.528	1.720	0.000	0.113	0.140
	GSE36527_CD62L_HIGH_CD69_NEG_VS_CD62L_LOW_CD69_POS_TREG_KLRG1_NEG_DN	0.590	1.712	0.000	0.114	0.140
	GSE45365_CD8A_DC_VS_CD11B_DC_IFNAR_KO_MCMV_INFECTION_DN	0.674	1.711	0.000	0.112	0.140
BCD.ID.SP	GSE22935_WT_VS_MYD88_KO_MACROPHAGE_UP	0.406	1.558	0.000	1.000	0.460
	GSE22886_NAIVE_BCELL_VS_MONOCYTE_DN	0.470	1.543	0.000	1.000	0.650
	GSE33425_CD161_INT_VS_NEG_CD8_TCELL_DN	0.410	1.525	0.000	1.000	0.650
	GSE22886_NAIVE_CD4_TCELL_VS_MONOCYTE_DN	0.491	1.523	0.000	1.000	0.650
	GSE22501_PERIPHERAL_BLOOD_VS_CORD_BLOOD_TREG_DN	0.440	1.520	0.000	1.000	0.650
	GSE3039_ALPHAALPHA_CD8_TCELL_VS_B2_BCELL_UP	0.449	1.519	0.000	1.000	0.650
	GSE27786_CD8_TCELL_VS_NKCELL_DN	0.406	1.518	0.000	1.000	0.650
	GSE10325_LUPUS_CD4_TCELL_VS_LUPUS_MYELOID_DN	0.492	1.506	0.000	1.000	0.650
	GSE34156_UNTREATED_VS_6H_TLR1_TLR2_LIGAND_TREATED_MONOCYTE_UP	0.511	1.504	0.000	1.000	0.650
	GSE22886_NAIVE_BCELL_VS_NEUTROPHIL_DN	0.502	1.504	0.000	1.000	0.650
BCD.IP.SP	GSE40274_CTRL_VS_XBP1_TRANSDUCED_ACTIVATED_CD4_TCELL_DN	0.463	1.715	0.000	0.602	0.170
	GSE15930_NAIVE_VS_48H_IN_VITRO_STIM_CD8_TCELL_DN	0.502	1.696	0.000	0.482	0.290
	GSE14415_INDUCED_TREG_VS_FAILED_INDUCED_TREG_UP	0.519	1.672	0.000	0.403	0.290
	GSE5142_HTERT_TRANSDUCED_VS_CTRL_CD8_TCELL_LATE_PASSAGE_CLONE_UP	0.438	1.662	0.000	0.333	0.290
	GSE3982_BASOPHIL_VS_TH1_DN	0.450	1.658	0.000	0.303	0.290
	GSE24142_ADULT_VS_FETAL_EARLY_THYMIC_PROGENITOR_DN	0.472	1.642	0.000	0.293	0.350
	GSE34156_UNTREATED_VS_24H_NOD2_AND_TLR1_TLR2_LIGAND_TREATED_MONOCYTE_UP	0.373	1.611	0.000	0.326	0.380
	GSE37301_LYMPHOID_PRIMED_MPP_VS_PRO_BCELL_DN	0.449	1.609	0.000	0.301	0.380
	GSE21360_SECONDARY_VS_TERTIARY_MEMORY_CD8_TCELL_UP	0.411	1.608	0.000	0.281	0.380
	KAECH_DAY8_EFF_VS_DAY15_EFF_CD8_TCELL_UP	0.397	1.578	0.000	0.347	0.450

Sample	GSEA Gene Sets	ES	NES	NOM p-val	FDR q-val	FWER p-val
Pre-Placebo	GSE21546_UNSTIM_VS_ANTI_CD3_STIM_ELK1_KO_DP_THYMOCYTES_UP	0.633	2.170	0.000	0.020	0.020
	GSE42021_CD24INT_VS_CD24LOW_TREG_THYMUS_DN	0.571	2.078	0.000	0.020	0.020
	GSE42021_CD24HI_VS_CD24LOW_TREG_THYMUS_DN	0.595	2.075	0.000	0.020	0.020
	GSE37533_PPARG1_FOXP3_VS_FOXP3_TRANSDUCED_CD4_TCELL_PIOGLITAZONE_TREATED_UP	0.607	2.028	0.000	0.020	0.020
	GSE6269_FLU_VS_STREP_PNEUMO_INF_PBMC_UP	0.638	1.985	0.000	0.020	0.020
	GSE21546_UNSTIM_VS_ANTI_CD3_STIM_SAP1A_KO_AND_ELK1_KO_DP_THYMOCYTES_UP	0.512	1.966	0.000	0.021	0.030
	GSE21546_ELK1_KO_VS_SAP1A_KO_AND_ELK1_KO_DP_THYMOCYTES_UP	0.612	1.962	0.000	0.021	0.030
	GSE42021_TREG_PLN_VS_CD24LO_TREG_THYMUS_DN	0.580	1.956	0.000	0.021	0.030
	GSE42021_CD24INT_VS_CD24LOW_TCONV_THYMUS_DN	0.498	1.950	0.000	0.022	0.030
Post-Placebo	GSE16385_IFNG_TNF_VS_UNSTIM_MACROPHAGE_ROSIGLITAZONE_TREATED_UP	0.535	2.162	0.000	0.010	0.000
	GSE42021_CD24HI_VS_CD24INT_TREG_THYMUS_DN	0.667	2.147	0.000	0.010	0.010
	GSE21546_UNSTIM_VS_ANTI_CD3_STIM_SAP1A_KO_AND_ELK1_KO_DP_THYMOCYTES_UP	0.615	2.133	0.000	0.010	0.010
	GSE42021_TREG_PLN_VS_CD24LO_TREG_THYMUS_DN	0.624	2.109	0.000	0.010	0.010
	GSE42724_NAIVE_BCELL_VS_PLASMABLAST_UP	0.739	2.102	0.000	0.010	0.010
	GSE13485_PRE_VS_POST_YF17D_VACCINATION_PBMC_DN	0.645	2.098	0.000	0.010	0.010
	GSE17974_IL4_AND_ANTI_IL12_VS_UNTREATED_24H_ACT_CD4_TCELL_DN	0.608	2.088	0.000	0.010	0.010
	GSE13485_CTRL_VS_DAY3_YF17D_VACCINE_PBMC_DN	0.701	2.083	0.000	0.010	0.010
	GSE13485_DAY3_VS_DAY7_YF17D_VACCINE_PBMC_DN	0.722	2.070	0.000	0.010	0.010
	GSE8835_CD4_VS_CD8_TCELL_CLL_PATIENT_UP	0.530	2.066	0.000	0.010	0.010
	GSE34205_HEALTHY_VS_FLU_INF_INFANT_PBMC_DN	0.623	2.226	0.000	0.020	0.000
Pre-Vaccine	GSE42021_TREG_PLN_VS_CD24LO_TREG_THYMUS_DN	0.711	2.601	0.000	0.000	0.000
	GSE41978_ID2_KO_VS_BIM_KO_KLRG1_LOW_EFFECTOR_CD8_TCELL_UP	0.715	2.597	0.000	0.000	0.000
	GSE13485_CTRL_VS_DAY3_YF17D_VACCINE_PBMC_DN	0.745	2.560	0.000	0.000	0.000
	GSE10325_MYELOID_VS_LUPUS_MYELOID_DN	0.678	2.502	0.000	0.000	0.000
	GSE42021_CD24INT_VS_CD24LOW_TREG_THYMUS_DN	0.679	2.478	0.000	0.000	0.000
	GSE13485_CTRL_VS_DAY7_YF17D_VACCINE_PBMC_DN	0.789	2.474	0.000	0.000	0.000
	GSE42021_TREG_VS_TCONV_PLN_UP	0.758	2.468	0.000	0.000	0.000
	GSE21360_NAIVE_VS_QUATERNARY_MEMORY_CD8_TCELL_DN	0.696	2.438	0.000	0.000	0.000
	GSE13485_PRE_VS_POST_YF17D_VACCINATION_PBMC_DN	0.709	2.435	0.000	0.000	0.000
	GSE42021_CD24HI_VS_CD24LOW_TREG_THYMUS_DN	0.690	2.425	0.000	0.000	0.000
Post-Vaccine	GSE42021_TREG_PLN_VS_CD24LO_TREG_THYMUS_DN	0.721	2.513	0.000	0.000	0.000
	GSE34156_UNTREATED_VS_6H_NOD2_AND_TLR1_TLR2_LIGAND_TREATED_MONOCYTE_DN	0.765	2.498	0.000	0.000	0.000
	GSE37534_UNTREATED_VS_PIOGLITAZONE_TREATED_CD4_TCELL_PPARG1_AND_FOXP3_TRASDUCED_DN	0.749	2.470	0.000	0.000	0.000
	GSE6259_DEC205_POS_DC_VS_BCELL_DN	0.646	2.464	0.000	0.000	0.000
	GSE33424_CD161_INT_VS_NEG_CD8_TCELL_UP	0.697	2.462	0.000	0.000	0.000
	GSE42021_TREG_PLN_VS_CD24INT_TREG_THYMUS_DN	0.782	2.441	0.000	0.000	0.000
	GSE22196_HEALTHY_VS_OBESE_MOUSE_SKIN_GAMMADELTA_TCELL_UP	0.692	2.437	0.000	0.000	0.000
	GSE13485_PRE_VS_POST_YF17D_VACCINATION_PBMC_DN	0.791	2.436	0.000	0.000	0.000
	GSE42021_TREG_PLN_VS_TREG_PRECURSORS_THYMUS_DN	0.769	2.433	0.000	0.000	0.000
	GSE19888_ADENOSINE_A3R_INH_PRETREAT_AND_ACT_BY_A3R_VS_TCELL_MEMBRANES_ACT_MAST_CELL_UP	0.777	2.433	0.000	0.000	0.000

Sample	GSEA Gene Sets	ES	NES	NOM p-val	FDR q-val	FWER p-val
UCNR_before-Treatment.CO	GSE29164_CD8_TCELL_VS_CD8_TCELL_AND_IL12_TREATED_MELANOMA_DAY7_DN	0.560	2.083	0.000	0.022	0.010
	GSE18281_CORTICAL_VS_MEDULLARY_THYMOCYTE_UP	0.650	2.072	0.000	0.011	0.010
	GSE42021_TREG_PLN_VS_CD24INT_TREG_THYMUS_DN	0.665	2.060	0.000	0.011	0.010
	GSE42021_TREG_VS_TCONV_PLN_UP	0.651	2.051	0.000	0.008	0.010
	GSE18791_UNSTIM_VS_NEWCATSLE_VIRUS_DC_10H_DN	0.637	2.049	0.000	0.007	0.010
	GSE42021_CD24HI_VS_CD24INT_TREG_THYMUS_DN	0.657	2.046	0.000	0.006	0.010
	GSE35685_CD34POS_CD10NEG_CD62LPOS_VS_CD34POS_CD10POS_BONE_MARROW_DN	0.574	2.045	0.000	0.005	0.010
	GSE40685_TREG_VS_FOXP3_KO_TREG_PRECURSOR_UP	0.617	2.037	0.000	0.004	0.010
	GSE18791_UNSTIM_VS_NEWCATSLE_VIRUS_DC_18H_DN	0.528	2.033	0.000	0.004	0.010
	GSE42021_CD24HI_VS_CD24INT_TCONV_THYMUS_DN	0.532	2.028	0.000	0.003	0.010
UCNR_after-Treatment.CO	GSE40666_WT_VS_STAT1_KO_CD8_TCELL_WITH_IFNA_STIM_90MIN_DN	0.543	2.040	0.000	0.410	0.040
	GSE13547_2H_VS_12_H_ANTI_IGM_STIM_BCELL_UP	0.579	2.009	0.000	0.312	0.050
	GSE9988_LOW_LPS_VS_CTRL_TREATED_MONOCYTE_UP	0.632	1.993	0.000	0.242	0.060
	GSE17974_CTRL_VS_ACT_IL4_AND_ANTI_IL12_0.5H_CD4_TCELL_DN	0.491	1.983	0.000	0.216	0.060
	GSE9988_LPS_VS_VEHICLE_TREATED_MONOCYTE_UP	0.639	1.977	0.000	0.180	0.060
	GSE20754_WT_VS_TCF1_KO_MEMORY_CD8_TCELL_UP	0.435	1.968	0.000	0.167	0.070
	GSE43863_DAY6_EFF_VS_DAY150_MEM_TH1_CD4_TCELL_UP	0.515	1.964	0.000	0.147	0.070
	GSE14415_NATURAL_TREG_VS_TCONV_DN	0.580	1.946	0.000	0.158	0.070
	GSE42021_TREG_PLN_VS_CD24HI_TREG_THYMUS_UP	0.488	1.934	0.000	0.159	0.080
	GSE20715_0H_VS_48H_OZONE_TLR4_KO_LUNG_DN	0.517	1.930	0.000	0.152	0.080
UCR_before-Treatment.CO	GSE13485_DAY1_VS_DAY7_YF17D_VACCINE_PBMC_DN	0.553	2.213	0.000	0.000	0.000
	GSE22886_NAIVE_VS_IGG_IGA_MEMORY_BCELL_DN	0.460	2.145	0.000	0.000	0.000
	GSE1432_1H_VS_6H_IFNG_MICROGLIA_DN	0.543	2.134	0.000	0.000	0.000
	GSE29614_CTRL_VS_DAY7_TIV_FLU_VACCINE_PBMC_DN	0.603	2.134	0.000	0.000	0.000
	GSE1432_1H_VS_24H_IFNG_MICROGLIA_DN	0.589	2.133	0.000	0.000	0.000
	GOLDRATH_NAIVE_VS_EFF_CD8_TCELL_DN	0.537	2.117	0.000	0.000	0.000
	GSE29614_DAY3_VS_DAY7_TIV_FLU_VACCINE_PBMC_DN	0.561	2.099	0.000	0.000	0.000
	GSE42021_TREG_PLN_VS_CD24INT_TREG_THYMUS_DN	0.668	2.093	0.000	0.000	0.000
	GSE42021_CD24HI_VS_CD24LOW_TCONV_THYMUS_DN	0.605	2.091	0.000	0.000	0.000
	GSE29164_CD8_TCELL_VS_CD8_TCELL_AND_IL12_TREATED_MELANOMA_DAY7_DN	0.522	2.086	0.000	0.000	0.000
UCR_after-Treatment.CO	GSE36891_POLYIC_TLR3_VS_PAM_TLR2_STIM_PERITONEAL_MACROPHAGE_UP	0.522	1.859	0.000	0.924	0.160
	GSE42021_TREG_PLN_VS_CD24HI_TREG_THYMUS_UP	0.428	1.772	0.000	1.000	0.240
	GSE42021_TCONV_PLN_VS_TREG_PRECURSORS_THYMUS_DN	0.386	1.760	0.000	1.000	0.250
	GSE27434_WT_VS_DNMT1_KO_TREG_DN	0.429	1.757	0.000	0.830	0.250
	GSE9988_LPS_VS_VEHICLE_TREATED_MONOCYTE_UP	0.443	1.748	0.000	0.733	0.270
	GSE2706_UNSTIM_VS_2H_LPS_AND_R848_DC_DN	0.433	1.744	0.018	0.644	0.270
	GSE17974_2H_VS_72H_UNTREATED_IN_VITRO_CD4_TCELL_UP	0.388	1.737	0.000	0.596	0.290
	GSE46606_UNSTIM_VS_CD40L_IL2_IL5_1DAY_STIMULATED_IRF4HIGH_SORTED_BCELL_DN	0.403	1.730	0.020	0.562	0.300
	GSE17974_CTRL_VS_ACT_IL4_AND_ANTI_IL12_0.5H_CD4_TCELL_DN	0.432	1.728	0.000	0.510	0.300
	GSE42021_TREG_PLN_VS_CD24INT_TREG_THYMUS_UP	0.368	1.698	0.000	0.640	0.380

Supplementary Figure S15. GSEA analysis.

For samples used in Supplementary Figure S14, we report the top ten most enriched terms of C7 immunologic gene sets in GSEA. Data used for the GSEA analysis are the same as those used for ICEPOP analysis.
